# Crystal structures and Hirshfeld surface analyses of tetra­kis­(4,5-di­hydro­furan-2-yl)silane and tetra­kis­(4,5-di­hydro­furan-2-yl)germane

**DOI:** 10.1107/S2056989023003158

**Published:** 2023-04-14

**Authors:** Arnold Ressel, Anna Krupp, Carsten Strohmann

**Affiliations:** a Technische Universität Dortmund, Fakultät für Chemie und Chemische Biologie, Anorganische Chemie, Otto-Hahn-Strasse 6, 44227 Dortmund, Germany; University of Aberdeen, United Kingdom

**Keywords:** crystal structure, di­hydro­furanyl groups (DHF), Hirshfeld surface analysis, di­hydro­furylsilan, di­hydro­furylgermane

## Abstract

The title compounds Si(C_4_H_5_O)_4_ (**1**) and Ge(C_4_H_5_O)_4_ (**2**) are di­hydro­furyl compounds of silicon and germanium and are useful building blocks for the functionalization of these elements. Both structures crystallize in space group *P*2_1_/*n* in the monoclinic crystal system with two mol­ecules in the asymmetric unit: the Si and Ge atoms adopt slightly distorted tetra­hedral geometries, while the C_4_H_5_O moieties exhibit shallow envelope conformations. Through a Hirshfeld surface analysis of the structures, inter­actions within the crystal packing could be elucidated: compound **1** features a polymeric chain in the (101) plane *via* C—H⋯O hydrogen bonds whereas in **2** C—H⋯O hydrogen bonds create a polymeric chain in the (010) plane.

## Abstract 2

The crystal structures of a di­hydro­furylsilane and a di­hydro­furylgermane are reported. Hirshfeld surface analyses were performed to investigate the inter­molecular inter­actions.

## 1. Chemical context

The first di­hydro­furylsilanes (DHF) were prepared by Lukevits and co-workers in the 1980s (Gevorgyan *et al.*, 1989 ▸). The di­hydro­furyl substituent is a good leaving group for nucleophilic substitution on silicon and has therefore been further investigated since then (Lukevits *et al.*, 1993 ▸). Primarily carbon nucleophiles (*e.g.* lithium alkyls) can be used for substitution of the DHF groups (Lukevits *et al.*, 1993 ▸). Nitro­gen and oxygen nucleophiles (*e.g.* LiNEt_2_ or *t*-butanol) also serve to cleave the Si­–C(DHF) bond: this is an alternative way of introducing an Si—N bond into a compound compared to conventional synthesis methods using, for example, meth­oxy­silanes (Bauer & Strohmann, 2014 ▸). With an oxygen nucleophile such as pyrocatechol, penta­valent silicates can be synthesized (Tacke *et al.*, 1991 ▸, 1993 ▸). Hydrides, likewise, are useful for substitutions (*e.g*. LiAlH_4_) (Gevorgyan *et al.*, 1990 ▸, 1992 ▸). Thus, silanes can be synthesised in a very precise way (Lukevics *et al.*, 1985 ▸, 1997 ▸). Analogous to DHF silanes, it is also possible to form germanes with di­hydro­furyl substituents. The substitution of the DHF groups on germanium is possible *via* lithium alkyls or hydrides, similar to silanes (Lukevics *et al.*, 1985 ▸). The crystal structures of various di­hydro­furylsilanes such as bis­(4,5-di­hydro­furan-2-yl)(dimeth­yl)silane and (4,5-di­hydro­furan-2-yl)(meth­yl)di­phenyl­silane (Schmidt *et al.*, 2022 ▸) or tris­(4,5-di­hydro­furan-2-yl)(meth­yl)silane and tris(4,5-di­hydro­furan-2-yl)(phen­yl)silane (Krupp *et al.*, 2020 ▸) are already known. Here, we report the crystal structures of tetra­kis­(4,5-di­hydro­furan-2-yl)silane, Si(C_4_H_5_O)_4_ (**1**) and tetra­kis­(4,5-di­hydro­furan-2-yl)germane, Ge(C_4_H_5_O)_4_ (**2**) and their extended structures, which were investigated using a Hirshfeld surface analysis. The two compounds are already known in the literature (Lukevics *et al.*, 1984 ▸; Ertschak *et al.*, 1982 ▸). 
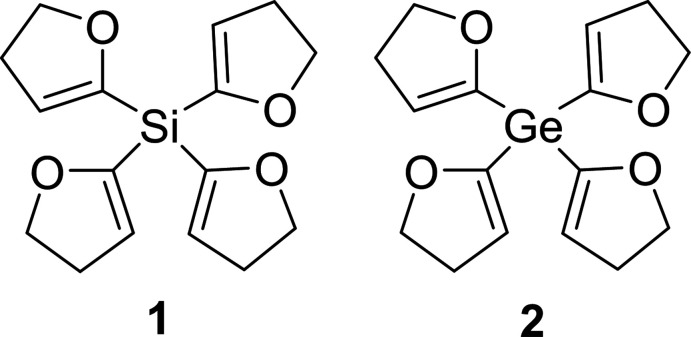


## 2. Structural commentary

**Figure 1:**
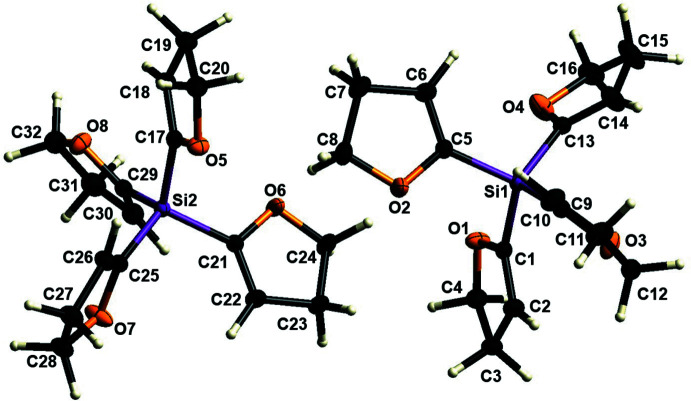
The mol­ecular structure of compound **1** with displacement ellipsoids drawn at the 50% probability level.

The mol­ecular structure of **1** is shown in Fig. 1 ▸ and selected bond lengths and angles of the solid-state structure are shown in Table 1 ▸. There are two mol­ecules in the asymmetric unit. The listed bond lengths of the Si–C(DHF) links are all in a comparable range. In addition, the bond lengths are consistent with the characteristic Si—C bond length (Allen *et al.*, 1987 ▸). The C—Si—C bond angles deviate from the ideal value of 109.47° and indicate a slightly distorted tetra­hedron. This has already been described in related compounds by Strohmann and co-workers (Krupp *et al.*, 2020 ▸; Schmidt *et al.*, 2022 ▸). This slight distortion is possibly due to the packing in the solid state. The bond lengths of the C=C bonds within the di­hydro­furanyl substituent show agreement with bond lengths known in the literature. The C—C single bond between the two *sp* ^3^ carbon atoms shows a clear deviation from the median known in literature, and is nearly in the lower quartile. This was also previously described by Strohmann and co-workers (Krupp *et al.*, 2020 ▸; Schmidt *et al.*, 2022 ▸). The DHF rings of the structure do not show complete planarity and have the r.m.s deviations shown in Table 2 ▸. The largest deviation of an atom from the planar position is shown by the *sp* ^3^ carbon atom C28, which is located next to the oxygen atom O7. This has also been reported for comparable structures (Schmidt *et al.*, 2022 ▸). In addition, Table 2 ▸ shows the dihedral angles between the normals of two rings.

**Figure 2:**
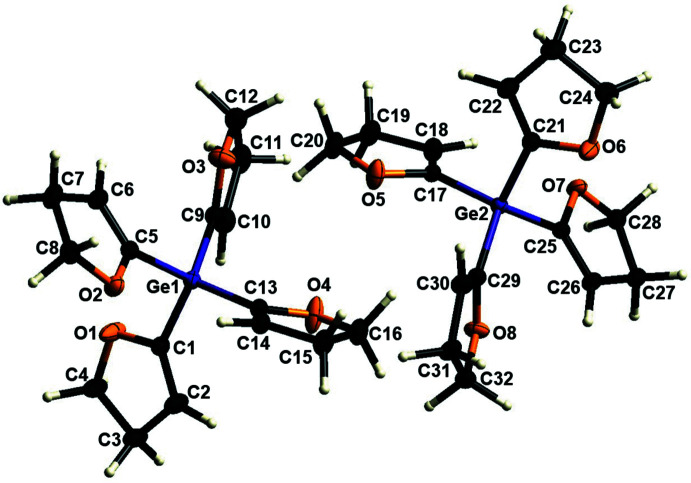
The mol­ecular structure of compound **2** with displacement ellipsoids drawn at the 50% probability level.

The mol­ecular structure of **2** is shown in Fig. 2 ▸. There are two mol­ecules in the asymmetric unit and selected bond lengths and angles are given in Table 3 ▸. The Ge—C bonds are in a comparable range and are consistent with similar bond lengths in the literature (Lazraq *et al.*, 1988 ▸). As already described for structure **1**, the germane **2** also shows a slight deviation from the ideal tetra­hedral value for the C—Ge—C bond angles, which can also be explained by the packing in the solid state. Likewise, the bond lengths of the C=C groups within the di­hydro­furanyl rings show consistency with bond lengths known in the literature, as well as the C—C bond between two *sp* ^3^ carbon atoms showing similar peculiarities as previously described (Krupp *et al.*, 2020 ▸; Schmidt *et al.*, 2022 ▸). The DHF rings of the structure do not show complete planarity and have the r.m.s deviations shown in Table 3 ▸. Compared to **1**, the deviations in **2** are smaller and, again, the *sp* ^3^ carbon atom C28, which is located next to O7, shows the highest deviation. However, this does not apply to the C21–C24/O6 ring as this has a very low r.m.s. deviation and C21 shows the highest deviation. The dihedral angles between the normals of two rings are listed in Table 4 ▸.

## 3. Supra­molecular features

**Figure 3:**
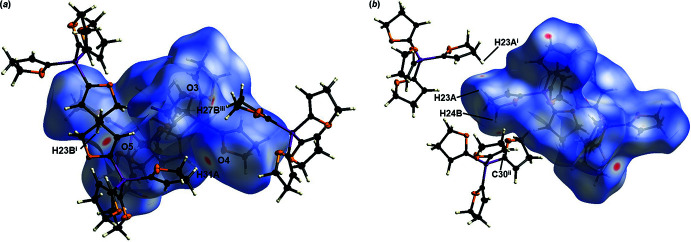
Hirshfeld surface analysis of **1** showing close contacts in the crystal. (*a*) The hydrogen bonds between C8—H8*A*⋯O5^ii^, C23^i^—H23*B* ^i^⋯O5, C27^iii^—H27*B* ^iii^⋯O3 and C31—H31*A*⋯O4 are labelled [symmetry codes: (i) − 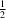 + *x*, 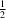 + *y*, 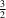 − *z*; (ii) 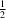 + *x*, 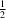 − *y*, 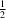 + *z*; (iii) 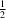 + *x*, 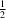 − *y*, 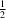 + *z*]. (*b*) The close hydrogen–hydrogen inter­action H23*A*⋯H23*A* ^i^ and close carbon–hydrogen inter­action C30^ii^⋯H24*B* are labelled [symmetry codes: (i) −*x* + 1, −*y* + 1, −*z* + 2; (ii) 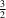 − *x*, − 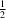 + *y*, 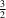 − *z*].

**Figure 4:**
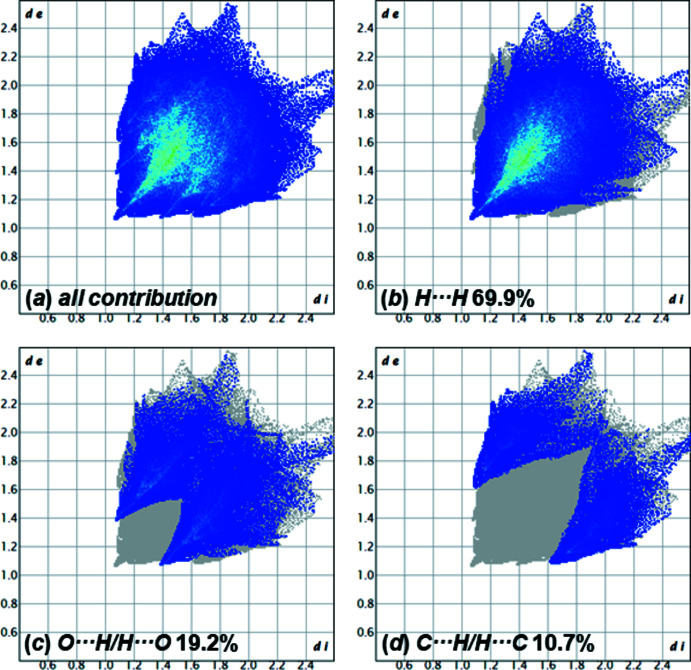
Two-dimensional fingerprint plots for compound **1**, showing (*a*) all contributions, and (*b*)–(*d*) delineated into the contributions of atoms within specific inter­acting pairs (blue areas).

**Figure 5:**
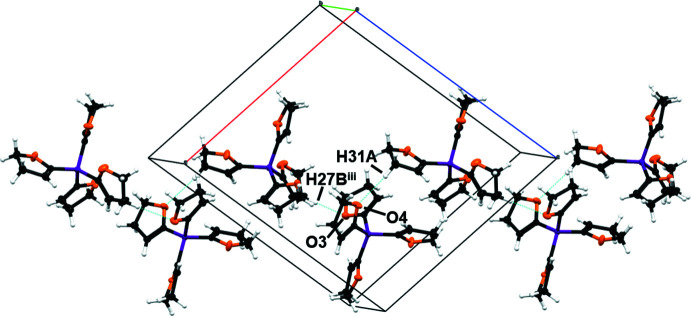
A part of the crystal packing of compound **1** *via* hydrogen bonds C27–H27*B*⋯O3^iii^ and C31—H31*A*⋯O4 in the (101) plane. C—H⋯O hydrogen bonds are shown as dashed blue lines. [Symmetry codes: (iii) − 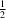 + *x*, 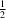 − *y*, 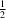 + *z*].

In order to qu­antify the inter­molecular inter­actions in the crystal structure, a Hirshfeld surface analysis was carried out, generated by *CrystalExplorer21* (Spackman *et al.*, 2021 ▸). The Hirshfeld surface of **1** is shown in Fig. 3 ▸, where the red areas represent closer inter­actions between adjacent atoms. The Hirshfeld surface is mapped over *d*_norm_, in the range −0.11 to 1.37 a.u. The distribution of the different inter­actions is illus­trated by the two-dimensional fingerprint plots (Fig. 4 ▸). Inter­actions identified by the Hirshfeld surface are mostly H⋯H inter­actions, which contribute 69.9% to the crystal packing. The close inter­action H23*A*⋯H23*A* ^i^ [symmetry code: (i) −*x*, −*y*, −*z*] with a distance of 2.22 (4) Å was identified by the red spots on the Hirshfeld surface. However, these red spots show only a small proportion of the inter­actions indicated in the fingerprint. Furthermore, a C30⋯H24*B* ^ii^ [symmetry code: (ii) 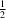 − *x*, 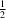 + *y*, 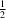 − *z*] van der Waals inter­action with a separation of 2.796 (16) Å was also identified. The contribution of the C⋯H inter­actions is 10.7%, which is a low contribution to the crystal packing. Besides these inter­actions, H⋯O inter­actions could be identified and contribute 19.2% of the structure in the solid state. Hydrogen bonds between C—H⋯O, which are indicated by red spots on the Hirshfeld surface are listed in Table 5 ▸. The C—H⋯O hydrogen bonds C8—H8*A*⋯O5^ii^, C27—H27*B*⋯O3^iii^ and C31—H31*A*⋯O4 can be described as having a *D*_1_^1^(2) graph-set motif. C23—H23*B*⋯O5^i^ is described by 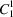 (7) (Etter *et al.*, 1990 ▸). Through the hydrogen bonds C27—H27*B*⋯O3^iii^ and C31—H31*A*⋯O4, a part of the crystal packing is defined along the [101] direction (Fig. 5 ▸).

**Figure 6:**
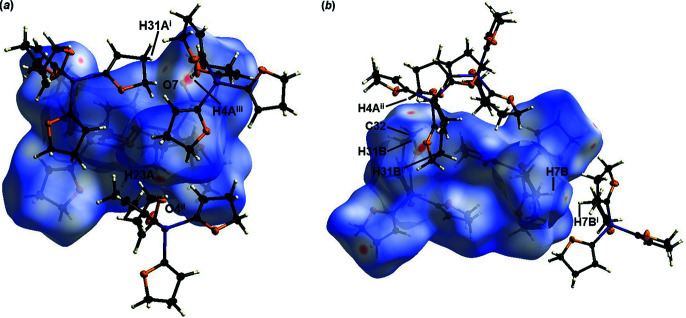
Hirshfeld surface analysis of **2** showing close contacts in the crystal. (*a*) The hydrogen bonds between C4^iii^—H4*A* ^iii^⋯O7, C23—H23*A*⋯O4^ii^ and C31^i^—H31*A* ^i^⋯O7 are labelled [symmetry codes: (i) 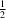 + *x*, − 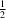 + *y*, 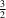 − *z*; (ii) − 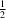 + *x*, 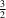 − *y*, 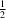 + *z*; (iii) *x*, *y*, 1 + *z*]. (*b*) The close hydrogen–hydrogen inter­actions H31*B*⋯H31*B* ^i^ and H7*B*⋯H7*B* ^i^ [symmetry code: (i) −*x*, −*y*, −*z*] and the close carbon–hydrogen inter­action C32⋯H4*A* ^ii^ are labelled [symmetry code: (ii) 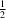 − *x*, 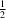 + *y*, 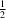 − *z*].

**Figure 7:**
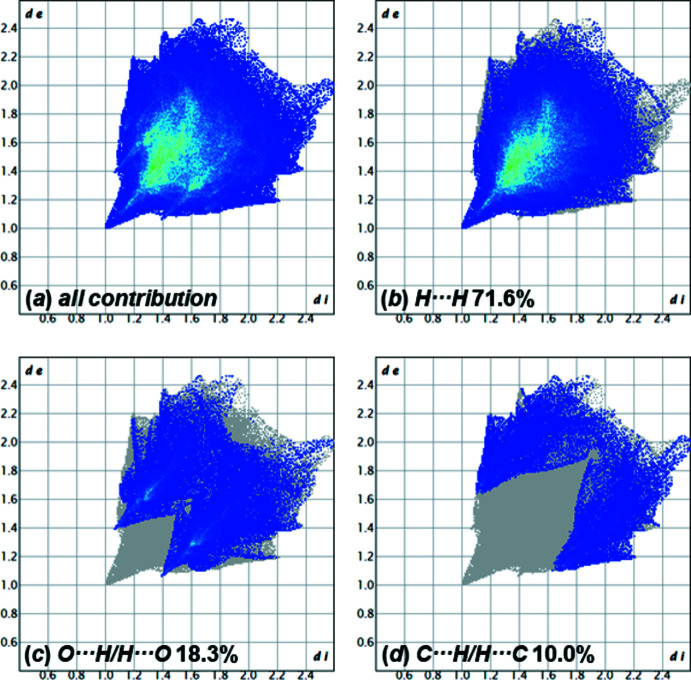
Two-dimensional fingerprint plots for compound **2**, showing (*a*) all contributions, and (*b*)–(*d*) delineated into the contributions of atoms within specific inter­acting pairs (blue areas).

**Figure 8:**
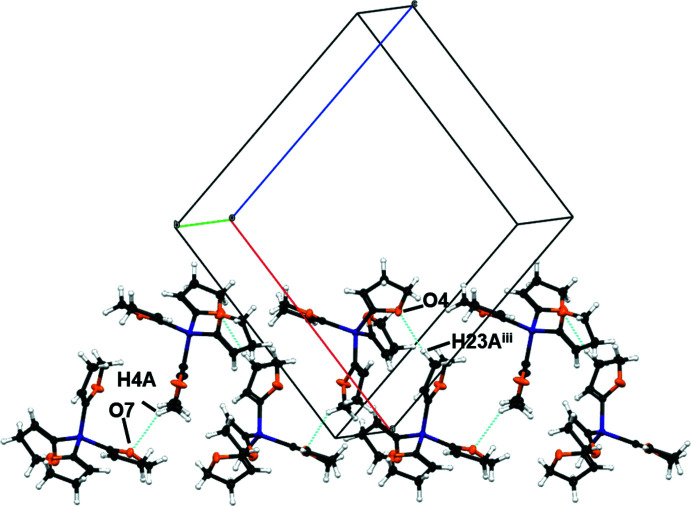
A part of the crystal packing of compound **2** *via* hydrogen bonds C4—H4*A*⋯O7^i^ and C23—H23*A*⋯O4^ii^ in the (010) plane. C—H⋯O hydrogen bonds are shown as dashed blue lines [symmetry codes: (i) 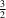 − *x*, − 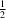 + *y*, 3/2 − z; (ii) − 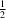 + *x*, 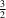 − *y*, 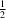 + *z*].

For the Hirshfeld surface analysis of **2**, a surface mapped over *d*_norm_ in the range −0.15 to 1.33 a.u. was used (Fig. 6 ▸). The distribution of the various inter­actions is illustrated by the two-dimensional fingerprint plots (Fig. 7 ▸). The distribution of the inter­actions is very similar and minimally larger for H⋯H (71.6%) than for **1**. The inter­actions between H31*B*⋯H31*B* ^i^ at 2.17 (4) Å and H7*B*⋯H7*B* ^i^ [symmetry code: (i) −*x*, −*y*, −*z*] at 2.20 (4) Å are visible as red spots and could be identified as close inter­actions by the Hirshfeld surface. The contribution of the van der Waals inter­actions is slightly lower at 10.0%. The inter­action C32⋯H4*A* ^ii^ [symmetry code: (ii) 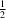 − *x*, 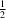 + *y*, 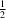 − *z*] at 2.79 (2) Å could also be detected on the Hirshfeld surface. As in the case of **1**, inter­actions of the form H⋯O could be determined, which contribute 18.3% to the crystal packing. These hydrogen bonds were detected by red spots on the Hirshfeld surface and are shown in Table 6 ▸. C4—H4*A*⋯O7^i^ and C23–H23*A*⋯O4^ii^ can be described by the graph-set motif *D*_1_^1^(2). In contrast, the hydrogen bond C31—H31*A*⋯O7^iii^ is described by the graph-set motif 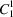 (7) (Etter *et al.*, 1990 ▸). With C4—H4*A*⋯O7^iii^ and C31—H31*A*⋯O7^i^, a part of the crystal packing, which forms a plane in the [010] direction, can be seen in Fig. 8 ▸.

## 4. Database survey

A search of the Cambridge Crystallographic Database (Groom *et al.*, 2016 ▸; WebCSD, accessed January 2023) for the term 2-(4,5-di­hydro­fur­yl)silanes gave bis­(4,5-di­hydro­furan-2-yl)(dimeth­yl)silane and (4,5-di­hydro­furan-2-yl)(meth­yl)di­phenyl­silane (CSD refcodes GAVJUM and GAVKAT; Schmidt *et al.*, 2022 ▸) as well as tris­(4,5-di­hydro­furan-2-yl)(meth­yl)silane and tris­(4,5-di­hydro­furan-2-yl)(phen­yl)silane (YUYCED and YUYCON; Krupp *et al.*, 2020 ▸), previously published by our group. These compounds show comparable Si—C(DHF), C—C and C=C bond lengths to those of **1** and **2**. They also display similar (DHF)C—Si—C(DHF) bond angles and also a slightly distorted tetra­hedron. In addition, a deviation in the planarity of the di­hydro­furyl rings was found there. An extended search for 3-(4,5-di­hydro­fur­yl)silanes revealed the compounds [4-(4-fluoro­phen­yl)-5-(4-nitro­phen­yl)-4,5-di­hydro­furan-3-yl](trimeth­yl)silane (JIVLIM; Li & Zhang, 2018 ▸), (1′*S*,2*R*)-5-methyl-4-(*t*-butyl­diphenyl­sil­yl)-2,3-di­hydro-furan-2-carb­oxy­lic acid (1′-phenyl­eth­yl)amide (PUXCAM; Evans *et al.*, 2001 ▸) and 2,2-di­chloro-5-phenyl-4-(tri­methyl­sil­yl)-3(2*H*)-furan­one (YIHDOI; Murakami *et al.*, 1994 ▸), which have little resemblance to the structure of **1**. Tetra­kis(2-furan­yl)silane was also found in the database when searching for (2-furan­yl)silane (XAMZOA; Neugebauer *et al.*, 2000 ▸).

A search for 2-(4,5-di­hydro­fur­yl)germane and an extended search for 3-(4,5-di­hydro­fur­yl)germane found no hits.

## 5. Synthesis and crystallization

Compound **1** and **2** have already been described by Lukevits and Ertschak (Lukevics *et al.*, 1984 ▸; Ertschak *et al.*, 1982 ▸). For the synthesis of tetra­kis­(4,5-di­hydro­furan-2-yl)silane (**1**), *tert*-butyl­lithium (31.0 ml, 1.90 *M* in pentane, 58.90 mmol, 4.00 eq.) was added at 228 K to a solution of 2,3-di­hydro­furan (4.14 g, 59.10 mmol, 4.00 eq.) in diethyl ether (approx. 100 ml). The reaction solution was stirred for 1 h at room temperature. Then, tetra­chloro­silane (2.50 g, 14.70 mmol, 1.00 eq.) was added at 243 K and the reaction solution was stirred for 1 h. The resulting solid was separated by inert filtration. The obtained solution was concentrated *in vacuo* and crystallized at 243 K. The solvent was removed and the solid was washed with cold diethyl ether. The product tetra­kis­(4,5-di­hydro­furan-2-yl)silane (**1**) (3.05 g, 10.0 mmol, 68%) was obtained as colourless blocks.

^1^H NMR: (600.29 MHz, C_6_D_6_): δ = 2.25 [*dt*, ^3^ *J*_HH_ = 2.57 Hz, ^3^ *J*_HH_ = 9.72 Hz, 8H; Si(CCHC*H*_2_)_4_], 4.06 [*t*, ^3^ *J*_HH_ = 9.72 Hz, 8H; Si(COC*H*_2_)_4_], 5.88 [*t*, ^3^ *J*_HH_ = 2.57 Hz, 4H; Si(CC*H*)_4_] ppm. {^1^H}^13^C NMR: (150.94 MHz, C_6_D_6_): δ = 31.4 [4C; Si(CCH*C*H_2_)_4_], 71.0 [4C; Si(CO*C*H_2_)_4_], 117.8 [4C; Si(C*C*H)_4_], 155.1 [4C; (Si(*C*O)_4_] ppm. {^1^H}^29^Si NMR: (119.26 MHz, C_6_D_6_): δ = −51.40 [s, 1Si; *Si*(DHF)_4_] ppm.

For the synthesis of tetra­kis­(4,5-di­hydro­furan-2-yl)germane (**2**), *tert*-butyl­lithium (19.60 ml, 1.90 M in pentane, 37.30 mmol, 4.00 eq.) was added at 228 K to a solution of 2,3-di­hydro­furan (2.60 g, 37.30 mmol, 4.00 eq.) in diethyl ether (approx. 100 ml). The reaction solution was stirred for 1 h at rt. Tetra­chloro­germane (2.00 g, 9.33 mmol, 1.00 eq.) was added at 213 K and the reaction solution was stirred for 1 h. The resulting solid was separated by inert filtration. The obtained solution was concentrated *in vacuo* and crystallized at 243 K. The solvent was removed, and the solid was washed with cold diethyl ether. The product tetra­kis­(4,5-di­hydro­furan-2-yl)germane (**2**) (2.94 g, 8.44 mmol, 91%) was obtained as colourless blocks.

^1^H NMR: (400.25 MHz, C_6_D_6_): δ = 2.26 [*dt*, ^3^ *J*_HH_ = 2.57 Hz, ^3^ *J*_HH_ = 9.66 Hz, 8H; Ge(CCHC*H*_2_)_4_], 4.05 [*t*, ^3^ *J*_HH_ = 9.66 Hz, 8H; Ge(COC*H*_2_)_4_], 5.62 [*t*, ^3^ *J*_HH_ = 2.57 Hz, 4H; Ge(CC*H*)_4_] ppm. {^1^H}^13^C NMR: (100.64 MHz, C_6_D_6_): δ = 30.8 [4C; Ge(CCH*C*H_2_)_4_], 71.0 [4C; Ge(CO*C*H_2_)_4_], 113.8 [4C; Si(C*C*H)_4_], 155.7 [4C; (Ge(*C*O)_4_] ppm.

## 6. Refinement

Crystal data, data collection and structure refinement details are summarized in Table 7 ▸. Hydrogen atoms H8*A*,*B*, H23*A*,*B*, H24*A*,*B*, H27*A*,*B* and H31*A*,*B* for compound **1** and all H atoms for compound **2** were refined independently. Other H atoms were positioned geometrically (C—H = 0.95–1.00 Å) and were refined using a riding model, with *U*_iso_(H) = 1.2*U*_eq_(C) for CH_2_ and CH hydrogen.

## Supplementary Material

Crystal structure: contains datablock(s) 1. DOI: 10.1107/S2056989023003158/hb8058sup1.cif

Structure factors: contains datablock(s) 1. DOI: 10.1107/S2056989023003158/hb80581sup2.hkl

Supporting information file. DOI: 10.1107/S2056989023003158/hb80581sup4.cml

Structure factors: contains datablock(s) 2. DOI: 10.1107/S2056989023003158/hb80582sup3.hkl

Supporting information file. DOI: 10.1107/S2056989023003158/hb80582sup5.cml

CCDC reference: 2254180

Additional supporting information:  crystallographic information; 3D view; checkCIF report

## Figures and Tables

**Table 1:** Selected geometric parameters (Å, °) for **1** [Chem scheme1]

Si1—C1	1.8632 (10)	Si2—C17	1.8621 (10)
Si1—C5	1.8611 (9)	Si2—C21	1.8598 (10)
Si1—C9	1.8604 (10)	Si2—C25	1.8615 (10)
Si1—C13	1.8633 (10)	Si2—C29	1.8609 (10)
			
C5—Si1—C1	110.75 (4)	C21—Si2—C17	106.75 (4)
C5—Si1—C13	108.60 (4)	C21—Si2—C25	109.54 (4)
C9—Si1—C1	109.39 (4)	C21—Si2—C29	112.87 (4)
C9—Si1—C5	106.46 (4)	C25—Si2—C17	110.57 (4)
C9—Si1—C13	113.04 (4)	C29—Si2—C17	108.47 (4)
C13—Si1—C1	108.60 (4)	C29—Si2—C25	108.64 (4)

**Table 2:** Conformations (Å, °) of the DHF rings for compound **1**

DHF ring	r.m.s. deviation	Largest deviation	Angle between ring normals
C1–C4/O1	0.067	C4 −0.0936 (6)	—
C5–C8/O2	0.038	C8 −0.0521 (8)	82.18 (4)^*a*^
C9–C12/O3	0.034	C12 −0.0468 (7)	42.32 (4)^*a*^
C13–C16/O4	0.049	C16 −0.0679 (7)	45.01 (4)^*a*^
C17–C20/O5	0.054	C20 −0.0934 (6)	—
C21–C24/O6	0.050	C24 −0.0699 (7)	48.41 (6)^*b*^
C25–C28/O7	0.093	C28 0.1298 (9)	55.30 (6)^*b*^
C29–C32/O8	0.029	C32 −0.0397 (7)	81.77 (4)^*b*^

Notes: (*a*) compared to C1–C4/O1; (*b*) compared to C17–C20/O5.

**Table 3:** Selected geometric parameters (Å, °) for **2** [Chem scheme1]

Ge1—C1	1.9331 (13)	Ge2—C17	1.9370 (13)
Ge1—C5	1.9326 (14)	Ge2—C21	1.9290 (13)
Ge1—C9	1.9299 (14)	Ge2—C25	1.9329 (13)
Ge1—C13	1.9351 (14)	Ge2—C29	1.9353 (14)
			
C1—Ge1—C13	109.37 (6)	C21—Ge2—C17	108.03 (6)
C5—Ge1—C1	109.74 (6)	C21—Ge2—C25	108.58 (5)
C5—Ge1—C13	108.78 (6)	C21—Ge2—C29	112.88 (6)
C9—Ge1—C1	107.67 (6)	C25—Ge2—C17	107.92 (5)
C9—Ge1—C5	108.46 (6)	C25—Ge2—C29	106.91 (6)
C9—Ge1—C13	112.79 (6)	C29—Ge2—C17	112.36 (6)

**Table 4:** Conformations (Å, °) of the DHF rings for compound **2**

DHF ring	r.m.s. deviation	Largest deviation	Angle between ring normals
C1–C4/O1	0.015	C3 −0.0196 (12)	—
C5–C8/O2	0.038	C8 −0.0526 (9)	81.75 (6)^*a*^
C9–C12/O3	0.017	C12 0.0240 (13)	62.38 (7)^*a*^
C13–C16/O4	0.029	C16 0.0399 (11)	82.47 (7)^*a*^
C17–C20/O5	0.032	C20 0.0442 (11)	—
C21–C24/O6	0.007	C21 0.0094 (10)	87.22 (9)^*b*^
C25–C28/O7	0.068	C28 −0.0941 (9)	45.36 (6)^*b*^
C29–C32/O8	0.033	C32 0.0462 (9)	80.68 (7)^*b*^

Notes: (*a*) compared to C1–C4/O1; (*b*) compared to C17–C20/O5.

**Table 5:** Hydrogen-bond geometry (Å, °) for **1** [Chem scheme1]

*D*—H⋯*A*	*D*—H	H⋯*A*	*D*⋯*A*	*D*—H⋯*A*
C23—H23*B*⋯O5^i^	0.974 (18)	2.531 (18)	3.3484 (14)	141.5 (14)
C8—H8*A*⋯O5^ii^	0.95 (2)	2.61 (2)	3.3800 (15)	137.9 (15)
C27—H27*B*⋯O3^iii^	0.92 (2)	2.61 (2)	3.4200 (16)	147.1 (18)
C31—H31*A*⋯O4	0.986 (18)	2.561 (18)	3.5358 (14)	169.6 (15)

Symmetry codes: (i) 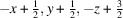 ; (ii) 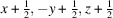 ; (iii) 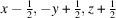 .

**Table 6:** Hydrogen-bond geometry (Å, °) for **2** [Chem scheme1]

*D*—H⋯*A*	*D*—H	H⋯*A*	*D*⋯*A*	*D*—H⋯*A*
C31—H31*A*⋯O7^i^	0.964 (19)	2.60 (2)	3.3279 (18)	132.1 (14)
C23—H23*A*⋯O4^ii^	0.97 (2)	2.57 (2)	3.530 (2)	168.0 (17)
C4—H4*A*⋯O7^iii^	0.93 (2)	2.63 (2)	3.398 (2)	140.8 (17)

Symmetry codes: (i) 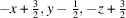 ; (ii) 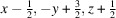 ; (iii) 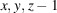 .

**Table 7:** Experimental details

	**1**	**2**
Crystal data		
Chemical formula	C_16_H_20_O_4_Si	C_16_H_20_GeO_4_
*M*_r_	304.41	348.91
Crystal system, space group	Monoclinic, *P*2_1_/*n*	Monoclinic, *P*2_1_/*n*
Temperature (K)	100	100
*a*, *b*, *c* (Å)	14.2044 (7), 14.2458 (7), 15.4851 (8)	14.3828 (5), 14.2069 (5), 15.3594 (6)
β (°)	102.605 (2)	101.159 (1)
*V* (Å^3^)	3057.9 (3)	3079.13 (19)
*Z*	8	8
Radiation type	Mo *K*α	Mo *K*α
μ (mm^−1^)	0.17	2.00
Crystal size (mm)	0.68 × 0.54 × 0.48	0.19 × 0.16 × 0.08

Data collection		
Diffractometer	Bruker D8 VENTURE	Bruker D8 VENTURE
Absorption correction	Multi-scan (*SADABS*; Krause *et al.*, 2015 ▸)	Multi-scan (*SADABS*; Krause *et al.*, 2015 ▸)
*T*_min_, *T*_max_	0.532, 0.570	0.496, 0.568
No. of measured, independent and observed [*I* > 2σ(*I*)] reflections	371220, 9331, 8779	71639, 13541, 10143
*R*_int_	0.036	0.046
(sin θ/λ)_max_ (Å^−1^)	0.714	0.807

Refinement		
*R*[*F* ^2^ > 2σ(*F* ^2^)], *wR*(*F* ^2^), *S*	0.037, 0.103, 1.03	0.032, 0.077, 1.02
No. of reflections	9331	13541
No. of parameters	419	539
H-atom treatment	H atoms treated by a mixture of independent and constrained refinement	All H-atom parameters refined
Δρ_max_, Δρ_min_ (e Å^−3^)	0.89, −0.38	0.67, −0.56

Computer programs: *APEX2* and *SAINT* (Bruker, 2018 ▸), *SHELXS* (Sheldrick, 2008 ▸), *SHELXT* (Sheldrick, 2015*a* ▸), *SHELXL2019/2* (Sheldrick, 2015*b* ▸), *OLEX2* (Dolomanov *et al.*, 2009 ▸), *CrystalExplorer21* (Spackman *et al.*, 2021 ▸), *Mercury* (Macrae *et al.*, 2020 ▸) and *publCIF* (Westrip, 2010 ▸).

## supplementary crystallographic information

### Tetrakis(4,5-dihydrofuran-2-yl)silane (1) Crystal data

**Table:**

C_16_H_20_O_4_Si	*F*(000) = 1296
*M**_r_* = 304.41	*D*_x_ = 1.322 Mg m^−^^3^
Monoclinic, *P*2_1_/*n*	Mo *K*α radiation, λ = 0.71073 Å
*a* = 14.2044 (7) Å	Cell parameters from 9905 reflections
*b* = 14.2458 (7) Å	θ = 2.6–17.2°
*c* = 15.4851 (8) Å	µ = 0.17 mm^−^^1^
β = 102.605 (2)°	*T* = 100 K
*V* = 3057.9 (3) Å^3^	Block, white
*Z* = 8	0.68 × 0.54 × 0.48 mm

### Tetrakis(4,5-dihydrofuran-2-yl)silane (1) Data collection

**Table:**

Bruker D8 VENTURE diffractometer	9331 independent reflections
Radiation source: microfocus sealed X-ray tube, Incoatec Iµs	8779 reflections with *I* > 2σ(*I*)
HELIOS mirror optics monochromator	*R*_int_ = 0.036
Detector resolution: 10.4167 pixels mm^-1^	θ_max_ = 30.5°, θ_min_ = 2.1°
ω and φ scans	*h* = −20→20
Absorption correction: multi-scan (SADABS; Krause *et al.*, 2015)	*k* = −20→20
*T*_min_ = 0.532, *T*_max_ = 0.570	*l* = −22→22
371220 measured reflections	

### Tetrakis(4,5-dihydrofuran-2-yl)silane (1) Refinement

**Table:**

Refinement on *F*^2^	Primary atom site location: structure-invariant direct methods
Least-squares matrix: full	Hydrogen site location: mixed
*R*[*F*^2^ > 2σ(*F*^2^)] = 0.037	H atoms treated by a mixture of independent and constrained refinement
*wR*(*F*^2^) = 0.103	*w* = 1/[σ^2^(*F*_o_^2^) + (0.0578*P*)^2^ + 1.2587*P*] where *P* = (*F*_o_^2^ + 2*F*_c_^2^)/3
*S* = 1.03	(Δ/σ)_max_ = 0.001
9331 reflections	Δρ_max_ = 0.89 e Å^−^^3^
419 parameters	Δρ_min_ = −0.38 e Å^−^^3^
0 restraints	

### Tetrakis(4,5-dihydrofuran-2-yl)silane (1) Special details

**Table:**

Geometry. All esds (except the esd in the dihedral angle between two l.s. planes) are estimated using the full covariance matrix. The cell esds are taken into account individually in the estimation of esds in distances, angles and torsion angles; correlations between esds in cell parameters are only used when they are defined by crystal symmetry. An approximate (isotropic) treatment of cell esds is used for estimating esds involving l.s. planes.

### Tetrakis(4,5-dihydrofuran-2-yl)silane (1) Fractional atomic coordinates and isotropic or equivalent isotropic displacement parameters (Å^2^)

**Table:**

	*x*	*y*	*z*	*U*_iso_*/*U*_eq_	
Si1	0.74622 (2)	0.28841 (2)	0.75077 (2)	0.01509 (6)	
O1	0.93162 (5)	0.25935 (5)	0.85344 (5)	0.02192 (14)	
O2	0.66166 (7)	0.30739 (6)	0.89526 (5)	0.02813 (17)	
O3	0.72770 (6)	0.45724 (6)	0.65291 (5)	0.02471 (15)	
O4	0.68036 (6)	0.15675 (7)	0.61688 (6)	0.03055 (19)	
C1	0.86791 (7)	0.32761 (7)	0.81134 (6)	0.01724 (16)	
C2	0.90373 (7)	0.41431 (7)	0.82338 (7)	0.02177 (18)	
H2	0.871274	0.469549	0.798507	0.026*	
C3	1.00352 (8)	0.41171 (8)	0.88241 (7)	0.0249 (2)	
H3A	1.003889	0.437693	0.941784	0.030*	
H3B	1.050636	0.446387	0.855916	0.030*	
C4	1.02411 (7)	0.30597 (8)	0.88647 (7)	0.02419 (19)	
H4A	1.070235	0.289812	0.849205	0.029*	
H4B	1.052002	0.286370	0.948076	0.029*	
C5	0.67360 (7)	0.24575 (7)	0.82938 (6)	0.01708 (16)	
C6	0.63131 (8)	0.16312 (7)	0.83393 (7)	0.02348 (19)	
H6	0.630973	0.112660	0.793750	0.028*	
C7	0.58398 (9)	0.16119 (7)	0.91211 (8)	0.0264 (2)	
H7A	0.616413	0.116278	0.957809	0.032*	
H7B	0.514695	0.144991	0.893973	0.032*	
C8	0.59827 (10)	0.26230 (8)	0.94469 (8)	0.0310 (2)	
C9	0.67957 (7)	0.39005 (7)	0.69126 (6)	0.01753 (16)	
C10	0.58506 (7)	0.40639 (8)	0.68000 (7)	0.02276 (18)	
H10	0.540808	0.367188	0.701030	0.027*	
C11	0.55907 (8)	0.49611 (8)	0.62906 (8)	0.0270 (2)	
H11A	0.537713	0.544989	0.666015	0.032*	
H11B	0.507913	0.485739	0.575246	0.032*	
C12	0.65508 (8)	0.52240 (8)	0.60570 (7)	0.0257 (2)	
H12A	0.649902	0.517179	0.541082	0.031*	
H12B	0.672832	0.587780	0.623997	0.031*	
C13	0.76134 (7)	0.19008 (7)	0.67562 (6)	0.01761 (16)	
C14	0.84148 (7)	0.14317 (7)	0.67128 (6)	0.02059 (17)	
H14	0.903761	0.156174	0.706378	0.025*	
C15	0.81904 (8)	0.06663 (8)	0.60272 (7)	0.02422 (19)	
H15A	0.827125	0.003588	0.630249	0.029*	
H15B	0.860033	0.071638	0.558782	0.029*	
C16	0.71307 (7)	0.08715 (8)	0.56085 (7)	0.02435 (19)	
H16A	0.706698	0.111915	0.500114	0.029*	
H16B	0.674129	0.029137	0.557871	0.029*	
Si2	0.27704 (2)	0.32421 (2)	0.76377 (2)	0.01562 (6)	
O5	0.09592 (6)	0.28846 (5)	0.65743 (5)	0.02374 (15)	
O6	0.27220 (6)	0.47644 (6)	0.87285 (5)	0.02643 (16)	
O7	0.21828 (7)	0.14802 (6)	0.81068 (5)	0.03273 (19)	
O8	0.34396 (7)	0.33545 (6)	0.60778 (5)	0.02971 (18)	
C17	0.15457 (7)	0.35882 (6)	0.70150 (6)	0.01701 (16)	
C18	0.11405 (8)	0.44363 (7)	0.69027 (7)	0.02251 (18)	
H18	0.143122	0.499966	0.716098	0.027*	
C19	0.01501 (8)	0.43670 (7)	0.63035 (7)	0.02359 (19)	
H19A	0.013165	0.466606	0.572297	0.028*	
H19B	−0.034782	0.465527	0.657760	0.028*	
C20	0.00210 (7)	0.33032 (7)	0.62152 (7)	0.02269 (19)	
H20A	−0.045780	0.308461	0.654906	0.027*	
H20B	−0.020752	0.312609	0.558609	0.027*	
C21	0.33102 (7)	0.42965 (7)	0.82634 (6)	0.01817 (16)	
C22	0.41603 (8)	0.47116 (8)	0.83225 (8)	0.0262 (2)	
H22	0.465781	0.449003	0.805262	0.031*	
C23	0.42186 (8)	0.55853 (8)	0.88805 (9)	0.0290 (2)	
C24	0.31965 (8)	0.56474 (7)	0.90322 (7)	0.02153 (18)	
C25	0.26895 (7)	0.22739 (7)	0.84255 (6)	0.01758 (16)	
C26	0.30693 (9)	0.22394 (8)	0.92899 (7)	0.0288 (2)	
H26	0.343000	0.272690	0.962848	0.035*	
C27	0.28359 (12)	0.13035 (9)	0.96465 (8)	0.0375 (3)	
C28	0.20576 (9)	0.09462 (8)	0.88849 (8)	0.0285 (2)	
H28A	0.213710	0.026545	0.879398	0.034*	
H28B	0.140908	0.105698	0.900266	0.034*	
C29	0.34752 (7)	0.28210 (7)	0.68322 (6)	0.01786 (16)	
C30	0.39575 (7)	0.20199 (7)	0.68338 (7)	0.02305 (19)	
H30	0.406776	0.157557	0.730256	0.028*	
C31	0.43036 (8)	0.19189 (8)	0.59856 (8)	0.0278 (2)	
C32	0.38774 (9)	0.28001 (8)	0.54776 (7)	0.0271 (2)	
H32A	0.439067	0.316510	0.528906	0.033*	
H32B	0.338577	0.262374	0.494472	0.033*	
H23A	0.4712 (13)	0.5512 (12)	0.9465 (11)	0.041 (5)*	
H23B	0.4341 (13)	0.6133 (13)	0.8545 (11)	0.041 (4)*	
H8A	0.6292 (14)	0.2656 (14)	1.0055 (13)	0.052 (5)*	
H8B	0.5353 (15)	0.2977 (14)	0.9324 (13)	0.053 (5)*	
H27A	0.3448 (15)	0.0826 (15)	0.9746 (13)	0.059 (6)*	
H27B	0.2664 (15)	0.1338 (15)	1.0187 (14)	0.061 (6)*	
H31A	0.5013 (13)	0.1894 (13)	0.6088 (11)	0.040 (4)*	
H31B	0.4046 (14)	0.1348 (14)	0.5651 (13)	0.050 (5)*	
H24A	0.3158 (10)	0.5717 (11)	0.9629 (10)	0.024 (3)*	
H24B	0.2840 (11)	0.6143 (11)	0.8672 (10)	0.030 (4)*	

### Tetrakis(4,5-dihydrofuran-2-yl)silane (1) Atomic displacement parameters (Å^2^)

**Table:**

	*U*^11^	*U*^22^	*U*^33^	*U*^12^	*U*^13^	*U*^23^
Si1	0.01493 (11)	0.01596 (12)	0.01465 (11)	−0.00030 (8)	0.00382 (8)	−0.00069 (8)
O1	0.0174 (3)	0.0222 (3)	0.0238 (3)	−0.0004 (3)	−0.0006 (3)	0.0016 (3)
O2	0.0447 (5)	0.0198 (3)	0.0264 (4)	−0.0128 (3)	0.0220 (3)	−0.0079 (3)
O3	0.0213 (3)	0.0232 (3)	0.0288 (4)	0.0003 (3)	0.0036 (3)	0.0100 (3)
O4	0.0163 (3)	0.0400 (5)	0.0336 (4)	0.0013 (3)	0.0016 (3)	−0.0198 (4)
C1	0.0162 (4)	0.0208 (4)	0.0150 (4)	−0.0006 (3)	0.0039 (3)	0.0003 (3)
C2	0.0225 (4)	0.0212 (4)	0.0208 (4)	−0.0030 (3)	0.0030 (3)	−0.0010 (3)
C3	0.0218 (4)	0.0290 (5)	0.0230 (4)	−0.0070 (4)	0.0026 (4)	−0.0047 (4)
C4	0.0167 (4)	0.0316 (5)	0.0227 (4)	−0.0027 (4)	0.0008 (3)	0.0011 (4)
C5	0.0181 (4)	0.0170 (4)	0.0168 (4)	−0.0005 (3)	0.0054 (3)	−0.0019 (3)
C6	0.0285 (5)	0.0179 (4)	0.0278 (5)	−0.0048 (4)	0.0145 (4)	−0.0059 (4)
C7	0.0351 (5)	0.0192 (4)	0.0295 (5)	−0.0081 (4)	0.0171 (4)	−0.0036 (4)
C8	0.0477 (7)	0.0216 (5)	0.0322 (6)	−0.0104 (5)	0.0271 (5)	−0.0062 (4)
C9	0.0191 (4)	0.0174 (4)	0.0159 (4)	−0.0001 (3)	0.0033 (3)	−0.0011 (3)
C10	0.0194 (4)	0.0243 (5)	0.0241 (4)	0.0021 (3)	0.0037 (3)	0.0000 (4)
C11	0.0244 (5)	0.0257 (5)	0.0277 (5)	0.0067 (4)	−0.0016 (4)	0.0003 (4)
C12	0.0290 (5)	0.0210 (4)	0.0246 (5)	0.0032 (4)	0.0000 (4)	0.0048 (4)
C13	0.0171 (4)	0.0190 (4)	0.0168 (4)	−0.0010 (3)	0.0037 (3)	−0.0020 (3)
C14	0.0198 (4)	0.0205 (4)	0.0201 (4)	0.0029 (3)	0.0012 (3)	−0.0025 (3)
C15	0.0240 (4)	0.0221 (4)	0.0252 (5)	0.0048 (4)	0.0025 (4)	−0.0058 (4)
C16	0.0217 (4)	0.0270 (5)	0.0240 (4)	−0.0009 (4)	0.0043 (3)	−0.0096 (4)
Si2	0.01722 (12)	0.01467 (12)	0.01523 (11)	−0.00029 (8)	0.00411 (9)	−0.00052 (8)
O5	0.0218 (3)	0.0164 (3)	0.0287 (4)	0.0020 (2)	−0.0038 (3)	−0.0029 (3)
O6	0.0260 (4)	0.0261 (4)	0.0300 (4)	−0.0089 (3)	0.0122 (3)	−0.0140 (3)
O7	0.0474 (5)	0.0228 (4)	0.0234 (4)	−0.0128 (3)	−0.0022 (3)	0.0023 (3)
O8	0.0418 (5)	0.0291 (4)	0.0225 (4)	0.0143 (3)	0.0164 (3)	0.0068 (3)
C17	0.0193 (4)	0.0165 (4)	0.0158 (4)	0.0002 (3)	0.0051 (3)	−0.0006 (3)
C18	0.0257 (5)	0.0171 (4)	0.0238 (4)	0.0023 (3)	0.0035 (4)	−0.0006 (3)
C19	0.0244 (4)	0.0227 (4)	0.0234 (4)	0.0074 (4)	0.0046 (4)	0.0032 (4)
C20	0.0178 (4)	0.0252 (5)	0.0245 (4)	0.0024 (3)	0.0033 (3)	−0.0003 (4)
C21	0.0218 (4)	0.0156 (4)	0.0174 (4)	−0.0007 (3)	0.0048 (3)	−0.0001 (3)
C22	0.0233 (5)	0.0203 (4)	0.0365 (5)	−0.0024 (4)	0.0100 (4)	−0.0042 (4)
C23	0.0243 (5)	0.0195 (4)	0.0425 (6)	−0.0054 (4)	0.0060 (4)	−0.0061 (4)
C24	0.0268 (5)	0.0170 (4)	0.0202 (4)	−0.0022 (3)	0.0037 (3)	−0.0028 (3)
C25	0.0187 (4)	0.0159 (4)	0.0183 (4)	0.0007 (3)	0.0044 (3)	0.0002 (3)
C26	0.0353 (6)	0.0249 (5)	0.0216 (5)	−0.0109 (4)	−0.0037 (4)	0.0042 (4)
C27	0.0539 (8)	0.0304 (6)	0.0227 (5)	−0.0157 (5)	−0.0039 (5)	0.0089 (4)
C28	0.0301 (5)	0.0211 (5)	0.0324 (5)	−0.0068 (4)	0.0025 (4)	0.0044 (4)
C29	0.0181 (4)	0.0186 (4)	0.0174 (4)	−0.0007 (3)	0.0049 (3)	−0.0005 (3)
C30	0.0219 (4)	0.0208 (4)	0.0283 (5)	0.0028 (3)	0.0095 (4)	0.0027 (4)
C31	0.0267 (5)	0.0268 (5)	0.0330 (5)	0.0044 (4)	0.0133 (4)	−0.0041 (4)
C32	0.0300 (5)	0.0330 (5)	0.0210 (4)	0.0036 (4)	0.0111 (4)	−0.0027 (4)

### Tetrakis(4,5-dihydrofuran-2-yl)silane (1) Geometric parameters (Å, º)

**Table:**

Si1—C1	1.8632 (10)	Si2—C17	1.8621 (10)
Si1—C5	1.8611 (9)	Si2—C21	1.8598 (10)
Si1—C9	1.8604 (10)	Si2—C25	1.8615 (10)
Si1—C13	1.8633 (10)	Si2—C29	1.8609 (10)
O1—C1	1.3901 (11)	O5—C17	1.3843 (11)
O1—C4	1.4613 (12)	O5—C20	1.4552 (12)
O2—C5	1.3842 (11)	O6—C21	1.3869 (12)
O2—C8	1.4525 (13)	O6—C24	1.4559 (12)
O3—C9	1.3834 (12)	O7—C25	1.3729 (12)
O3—C12	1.4598 (12)	O7—C28	1.4683 (14)
O4—C13	1.3853 (11)	O8—C29	1.3853 (12)
O4—C16	1.4587 (12)	O8—C32	1.4583 (13)
C1—C2	1.3334 (13)	C17—C18	1.3332 (13)
C2—H2	0.9500	C18—H18	0.9500
C2—C3	1.5102 (14)	C18—C19	1.5099 (15)
C3—H3A	0.9900	C19—H19A	0.9900
C3—H3B	0.9900	C19—H19B	0.9900
C3—C4	1.5332 (16)	C19—C20	1.5291 (15)
C4—H4A	0.9900	C20—H20A	0.9900
C4—H4B	0.9900	C20—H20B	0.9900
C5—C6	1.3304 (13)	C21—C22	1.3297 (14)
C6—H6	0.9500	C22—H22	0.9500
C6—C7	1.5078 (14)	C22—C23	1.5071 (15)
C7—H7A	0.9900	C23—C24	1.5230 (15)
C7—H7B	0.9900	C23—H23A	1.022 (18)
C7—C8	1.5251 (15)	C23—H23B	0.974 (18)
C8—H8A	0.95 (2)	C24—H24A	0.942 (14)
C8—H8B	1.01 (2)	C24—H24B	0.971 (16)
C9—C10	1.3358 (13)	C25—C26	1.3303 (14)
C10—H10	0.9500	C26—H26	0.9500
C10—C11	1.5049 (15)	C26—C27	1.5073 (16)
C11—H11A	0.9900	C27—C28	1.5175 (17)
C11—H11B	0.9900	C27—H27A	1.09 (2)
C11—C12	1.5317 (16)	C27—H27B	0.92 (2)
C12—H12A	0.9900	C28—H28A	0.9900
C12—H12B	0.9900	C28—H28B	0.9900
C13—C14	1.3343 (13)	C29—C30	1.3307 (13)
C14—H14	0.9500	C30—H30	0.9500
C14—C15	1.5065 (14)	C30—C31	1.5065 (15)
C15—H15A	0.9900	C31—C32	1.5341 (17)
C15—H15B	0.9900	C31—H31A	0.986 (18)
C15—C16	1.5315 (15)	C31—H31B	0.99 (2)
C16—H16A	0.9900	C32—H32A	0.9900
C16—H16B	0.9900	C32—H32B	0.9900
			
C5—Si1—C1	110.75 (4)	C21—Si2—C17	106.75 (4)
C5—Si1—C13	108.60 (4)	C21—Si2—C25	109.54 (4)
C9—Si1—C1	109.39 (4)	C21—Si2—C29	112.87 (4)
C9—Si1—C5	106.46 (4)	C25—Si2—C17	110.57 (4)
C9—Si1—C13	113.04 (4)	C29—Si2—C17	108.47 (4)
C13—Si1—C1	108.60 (4)	C29—Si2—C25	108.64 (4)
C1—O1—C4	106.85 (8)	C17—O5—C20	107.10 (7)
C5—O2—C8	107.31 (8)	C21—O6—C24	107.11 (8)
C9—O3—C12	107.07 (8)	C25—O7—C28	106.23 (8)
C13—O4—C16	107.27 (8)	C29—O8—C32	107.34 (8)
O1—C1—Si1	117.64 (7)	O5—C17—Si2	117.21 (7)
C2—C1—Si1	129.18 (8)	C18—C17—Si2	129.46 (8)
C2—C1—O1	113.14 (8)	C18—C17—O5	113.31 (9)
C1—C2—H2	125.0	C17—C18—H18	125.1
C1—C2—C3	109.94 (9)	C17—C18—C19	109.80 (9)
C3—C2—H2	125.0	C19—C18—H18	125.1
C2—C3—H3A	111.5	C18—C19—H19A	111.5
C2—C3—H3B	111.5	C18—C19—H19B	111.5
C2—C3—C4	101.21 (8)	C18—C19—C20	101.38 (8)
H3A—C3—H3B	109.3	H19A—C19—H19B	109.3
C4—C3—H3A	111.5	C20—C19—H19A	111.5
C4—C3—H3B	111.5	C20—C19—H19B	111.5
O1—C4—C3	106.46 (8)	O5—C20—C19	106.85 (8)
O1—C4—H4A	110.4	O5—C20—H20A	110.4
O1—C4—H4B	110.4	O5—C20—H20B	110.4
C3—C4—H4A	110.4	C19—C20—H20A	110.4
C3—C4—H4B	110.4	C19—C20—H20B	110.4
H4A—C4—H4B	108.6	H20A—C20—H20B	108.6
O2—C5—Si1	116.71 (7)	O6—C21—Si2	115.61 (7)
C6—C5—Si1	130.14 (7)	C22—C21—Si2	131.30 (8)
C6—C5—O2	113.14 (8)	C22—C21—O6	113.05 (9)
C5—C6—H6	124.9	C21—C22—H22	125.0
C5—C6—C7	110.13 (9)	C21—C22—C23	110.01 (9)
C7—C6—H6	124.9	C23—C22—H22	125.0
C6—C7—H7A	111.5	C22—C23—C24	101.63 (8)
C6—C7—H7B	111.5	C22—C23—H23A	111.4 (10)
C6—C7—C8	101.42 (8)	C22—C23—H23B	110.5 (10)
H7A—C7—H7B	109.3	C24—C23—H23A	111.2 (10)
C8—C7—H7A	111.5	C24—C23—H23B	108.8 (10)
C8—C7—H7B	111.5	H23A—C23—H23B	112.7 (14)
O2—C8—C7	107.24 (8)	O6—C24—C23	106.83 (8)
O2—C8—H8A	107.2 (12)	O6—C24—H24A	106.6 (9)
O2—C8—H8B	107.8 (11)	O6—C24—H24B	107.2 (9)
C7—C8—H8A	112.0 (12)	C23—C24—H24A	114.7 (9)
C7—C8—H8B	111.2 (12)	C23—C24—H24B	110.5 (9)
H8A—C8—H8B	111.2 (16)	H24A—C24—H24B	110.6 (13)
O3—C9—Si1	120.36 (7)	O7—C25—Si2	118.49 (7)
C10—C9—Si1	126.06 (8)	C26—C25—Si2	128.12 (8)
C10—C9—O3	113.58 (9)	C26—C25—O7	113.39 (9)
C9—C10—H10	125.0	C25—C26—H26	125.4
C9—C10—C11	109.94 (9)	C25—C26—C27	109.14 (10)
C11—C10—H10	125.0	C27—C26—H26	125.4
C10—C11—H11A	111.4	C26—C27—C28	101.14 (9)
C10—C11—H11B	111.4	C26—C27—H27A	111.9 (11)
C10—C11—C12	101.67 (8)	C26—C27—H27B	114.0 (14)
H11A—C11—H11B	109.3	C28—C27—H27A	108.9 (11)
C12—C11—H11A	111.4	C28—C27—H27B	115.5 (14)
C12—C11—H11B	111.4	H27A—C27—H27B	105.5 (17)
O3—C12—C11	107.13 (8)	O7—C28—C27	105.41 (9)
O3—C12—H12A	110.3	O7—C28—H28A	110.7
O3—C12—H12B	110.3	O7—C28—H28B	110.7
C11—C12—H12A	110.3	C27—C28—H28A	110.7
C11—C12—H12B	110.3	C27—C28—H28B	110.7
H12A—C12—H12B	108.5	H28A—C28—H28B	108.8
O4—C13—Si1	118.38 (7)	O8—C29—Si2	117.54 (7)
C14—C13—Si1	128.60 (7)	C30—C29—Si2	128.85 (8)
C14—C13—O4	112.99 (8)	C30—C29—O8	113.34 (9)
C13—C14—H14	124.9	C29—C30—H30	124.9
C13—C14—C15	110.26 (9)	C29—C30—C31	110.25 (9)
C15—C14—H14	124.9	C31—C30—H30	124.9
C14—C15—H15A	111.5	C30—C31—C32	101.63 (8)
C14—C15—H15B	111.5	C30—C31—H31A	112.3 (10)
C14—C15—C16	101.39 (8)	C30—C31—H31B	112.4 (11)
H15A—C15—H15B	109.3	C32—C31—H31A	112.8 (11)
C16—C15—H15A	111.5	C32—C31—H31B	110.1 (11)
C16—C15—H15B	111.5	H31A—C31—H31B	107.6 (15)
O4—C16—C15	106.81 (8)	O8—C32—C31	106.97 (8)
O4—C16—H16A	110.4	O8—C32—H32A	110.3
O4—C16—H16B	110.4	O8—C32—H32B	110.3
C15—C16—H16A	110.4	C31—C32—H32A	110.3
C15—C16—H16B	110.4	C31—C32—H32B	110.3
H16A—C16—H16B	108.6	H32A—C32—H32B	108.6
			
Si1—C1—C2—C3	176.08 (7)	Si2—C17—C18—C19	177.05 (7)
Si1—C5—C6—C7	177.67 (8)	Si2—C21—C22—C23	176.66 (8)
Si1—C9—C10—C11	179.51 (7)	Si2—C25—C26—C27	−178.46 (9)
Si1—C13—C14—C15	177.84 (8)	Si2—C29—C30—C31	172.10 (8)
O1—C1—C2—C3	−1.60 (12)	O5—C17—C18—C19	−0.92 (12)
O2—C5—C6—C7	−1.12 (13)	O6—C21—C22—C23	−0.58 (14)
O3—C9—C10—C11	−1.00 (12)	O7—C25—C26—C27	1.49 (15)
O4—C13—C14—C15	−0.17 (13)	O8—C29—C30—C31	−1.61 (13)
C1—Si1—C5—O2	55.68 (9)	C17—Si2—C21—O6	47.49 (8)
C1—Si1—C5—C6	−123.08 (10)	C17—Si2—C21—C22	−129.70 (11)
C1—Si1—C9—O3	36.98 (9)	C17—Si2—C25—O7	53.20 (9)
C1—Si1—C9—C10	−143.56 (9)	C17—Si2—C25—C26	−126.85 (11)
C1—Si1—C13—O4	−173.06 (8)	C17—Si2—C29—O8	45.79 (9)
C1—Si1—C13—C14	9.04 (11)	C17—Si2—C29—C30	−127.70 (10)
C1—O1—C4—C3	14.79 (10)	C17—O5—C20—C19	12.05 (11)
C1—C2—C3—C4	10.34 (11)	C17—C18—C19—C20	8.05 (11)
C2—C3—C4—O1	−14.89 (10)	C18—C19—C20—O5	−11.93 (10)
C4—O1—C1—Si1	173.47 (7)	C20—O5—C17—Si2	174.56 (6)
C4—O1—C1—C2	−8.56 (11)	C20—O5—C17—C18	−7.20 (11)
C5—Si1—C1—O1	68.57 (8)	C21—Si2—C17—O5	−172.97 (7)
C5—Si1—C1—C2	−109.02 (10)	C21—Si2—C17—C18	9.12 (11)
C5—Si1—C9—O3	156.69 (7)	C21—Si2—C25—O7	170.57 (8)
C5—Si1—C9—C10	−23.85 (10)	C21—Si2—C25—C26	−9.48 (12)
C5—Si1—C13—O4	66.43 (9)	C21—Si2—C29—O8	−72.29 (9)
C5—Si1—C13—C14	−111.48 (10)	C21—Si2—C29—C30	114.22 (10)
C5—O2—C8—C7	8.28 (14)	C21—O6—C24—C23	−11.72 (11)
C5—C6—C7—C8	5.96 (13)	C21—C22—C23—C24	−6.51 (13)
C6—C7—C8—O2	−8.43 (13)	C22—C23—C24—O6	10.84 (11)
C8—O2—C5—Si1	176.40 (8)	C24—O6—C21—Si2	−169.79 (7)
C8—O2—C5—C6	−4.63 (13)	C24—O6—C21—C22	7.92 (12)
C9—Si1—C1—O1	−174.39 (7)	C25—Si2—C17—O5	−53.90 (8)
C9—Si1—C1—C2	8.02 (11)	C25—Si2—C17—C18	128.20 (9)
C9—Si1—C5—O2	−63.14 (8)	C25—Si2—C21—O6	−72.25 (8)
C9—Si1—C5—C6	118.10 (10)	C25—Si2—C21—C22	110.57 (11)
C9—Si1—C13—O4	−51.47 (9)	C25—Si2—C29—O8	166.02 (8)
C9—Si1—C13—C14	130.62 (9)	C25—Si2—C29—C30	−7.47 (11)
C9—O3—C12—C11	7.40 (11)	C25—O7—C28—C27	−20.72 (13)
C9—C10—C11—C12	5.34 (12)	C25—C26—C27—C28	−13.98 (15)
C10—C11—C12—O3	−7.57 (11)	C26—C27—C28—O7	20.51 (14)
C12—O3—C9—Si1	175.37 (7)	C28—O7—C25—Si2	−167.63 (7)
C12—O3—C9—C10	−4.16 (12)	C28—O7—C25—C26	12.41 (13)
C13—Si1—C1—O1	−50.60 (8)	C29—Si2—C17—O5	65.13 (8)
C13—Si1—C1—C2	131.81 (9)	C29—Si2—C17—C18	−112.78 (10)
C13—Si1—C5—O2	174.85 (7)	C29—Si2—C21—O6	166.57 (7)
C13—Si1—C5—C6	−3.91 (12)	C29—Si2—C21—C22	−10.62 (12)
C13—Si1—C9—O3	−84.16 (8)	C29—Si2—C25—O7	−65.72 (9)
C13—Si1—C9—C10	95.30 (9)	C29—Si2—C25—C26	114.22 (11)
C13—O4—C16—C15	11.15 (12)	C29—O8—C32—C31	−7.05 (12)
C13—C14—C15—C16	6.83 (12)	C29—C30—C31—C32	−2.78 (12)
C14—C15—C16—O4	−10.65 (11)	C30—C31—C32—O8	5.86 (12)
C16—O4—C13—Si1	174.68 (7)	C32—O8—C29—Si2	−168.91 (7)
C16—O4—C13—C14	−7.10 (12)	C32—O8—C29—C30	5.58 (13)

### Tetrakis(4,5-dihydrofuran-2-yl)silane (1) Hydrogen-bond geometry (Å, º)

**Table:**

*D*—H···*A*	*D*—H	H···*A*	*D*···*A*	*D*—H···*A*
C23—H23*B*···O5^i^	0.974 (18)	2.531 (18)	3.3484 (14)	141.5 (14)
C8—H8*A*···O5^ii^	0.95 (2)	2.61 (2)	3.3800 (15)	137.9 (15)
C27—H27*B*···O3^iii^	0.92 (2)	2.61 (2)	3.4200 (16)	147.1 (18)
C31—H31*A*···O4	0.986 (18)	2.561 (18)	3.5358 (14)	169.6 (15)

Symmetry codes: (i) −*x*+1/2, *y*+1/2, −*z*+3/2; (ii) *x*+1/2, −*y*+1/2, *z*+1/2; (iii) *x*−1/2, −*y*+1/2, *z*+1/2.

### Tetrakis(4,5-dihydrofuran-2-yl)germane (2) Crystal data

**Table:**

C_16_H_20_GeO_4_	*F*(000) = 1440
*M**_r_* = 348.91	*D*_x_ = 1.505 Mg m^−^^3^
Monoclinic, *P*2_1_/*n*	Mo *K*α radiation, λ = 0.71073 Å
*a* = 14.3828 (5) Å	Cell parameters from 8295 reflections
*b* = 14.2069 (5) Å	θ = 2.6–23.2°
*c* = 15.3594 (6) Å	µ = 2.00 mm^−^^1^
β = 101.159 (1)°	*T* = 100 K
*V* = 3079.13 (19) Å^3^	Block, colourless
*Z* = 8	0.19 × 0.16 × 0.08 mm

### Tetrakis(4,5-dihydrofuran-2-yl)germane (2) Data collection

**Table:**

Bruker D8 VENTURE diffractometer	13541 independent reflections
Radiation source: microfocus sealed X-ray tube, Incoatec Iµs	10143 reflections with *I* > 2σ(*I*)
HELIOS mirror optics monochromator	*R*_int_ = 0.046
Detector resolution: 10.4167 pixels mm^-1^	θ_max_ = 35.0°, θ_min_ = 2.2°
ω and φ scans	*h* = −23→23
Absorption correction: multi-scan (SADABS; Krause *et al.*, 2015)	*k* = −22→22
*T*_min_ = 0.496, *T*_max_ = 0.568	*l* = −24→24
71639 measured reflections	

### Tetrakis(4,5-dihydrofuran-2-yl)germane (2) Refinement

**Table:**

Refinement on *F*^2^	Primary atom site location: dual
Least-squares matrix: full	Hydrogen site location: difference Fourier map
*R*[*F*^2^ > 2σ(*F*^2^)] = 0.032	All H-atom parameters refined
*wR*(*F*^2^) = 0.077	*w* = 1/[σ^2^(*F*_o_^2^) + (0.0339*P*)^2^ + 0.639*P*] where *P* = (*F*_o_^2^ + 2*F*_c_^2^)/3
*S* = 1.02	(Δ/σ)_max_ = 0.002
13541 reflections	Δρ_max_ = 0.67 e Å^−^^3^
539 parameters	Δρ_min_ = −0.56 e Å^−^^3^
0 restraints	

### Tetrakis(4,5-dihydrofuran-2-yl)germane (2) Special details

**Table:**

Geometry. All esds (except the esd in the dihedral angle between two l.s. planes) are estimated using the full covariance matrix. The cell esds are taken into account individually in the estimation of esds in distances, angles and torsion angles; correlations between esds in cell parameters are only used when they are defined by crystal symmetry. An approximate (isotropic) treatment of cell esds is used for estimating esds involving l.s. planes.

### Tetrakis(4,5-dihydrofuran-2-yl)germane (2) Fractional atomic coordinates and isotropic or equivalent isotropic displacement parameters (Å^2^)

**Table:**

	*x*	*y*	*z*	*U*_iso_*/*U*_eq_	
Ge1	0.76646 (2)	0.79656 (2)	0.25601 (2)	0.01725 (4)	
O1	0.85960 (11)	0.80852 (8)	0.11071 (9)	0.0405 (3)	
O2	0.57907 (7)	0.77199 (8)	0.15770 (7)	0.0234 (2)	
O3	0.78696 (8)	0.96600 (8)	0.35784 (8)	0.0284 (2)	
O4	0.82066 (8)	0.67821 (10)	0.40676 (8)	0.0360 (3)	
C1	0.83902 (10)	0.74624 (10)	0.17308 (9)	0.0198 (2)	
C2	0.87060 (12)	0.66001 (11)	0.16430 (11)	0.0266 (3)	
H2	0.8597 (14)	0.6081 (15)	0.2001 (13)	0.042 (6)*	
C3	0.91833 (13)	0.65498 (12)	0.08575 (12)	0.0284 (3)	
H3A	0.8873 (15)	0.6142 (16)	0.0419 (14)	0.046 (6)*	
H3B	0.9832 (15)	0.6336 (15)	0.1007 (13)	0.040 (6)*	
C4	0.91262 (16)	0.75656 (12)	0.05442 (12)	0.0337 (4)	
C5	0.64477 (10)	0.84006 (10)	0.19256 (9)	0.0192 (2)	
C6	0.61438 (11)	0.92783 (11)	0.17540 (10)	0.0240 (3)	
H6	0.6487 (14)	0.9854 (14)	0.1928 (12)	0.032 (5)*	
C7	0.51501 (11)	0.92654 (13)	0.12080 (11)	0.0289 (3)	
C8	0.49159 (10)	0.82158 (12)	0.11877 (10)	0.0250 (3)	
H8A	0.4679 (15)	0.7944 (14)	0.0582 (14)	0.038 (6)*	
H8B	0.4508 (13)	0.8053 (12)	0.1537 (12)	0.021 (4)*	
C9	0.83515 (10)	0.90293 (10)	0.31455 (9)	0.0200 (2)	
C10	0.92565 (11)	0.92402 (13)	0.31889 (11)	0.0288 (3)	
H10	0.9719 (16)	0.8885 (15)	0.2931 (14)	0.049 (6)*	
C11	0.94986 (12)	1.01363 (13)	0.37105 (12)	0.0319 (4)	
H11A	0.9997 (14)	1.0013 (14)	0.4282 (13)	0.039 (6)*	
H11B	0.9740 (13)	1.0595 (14)	0.3353 (12)	0.034 (5)*	
C12	0.85364 (12)	1.04082 (12)	0.39196 (11)	0.0280 (3)	
H12A	0.8284 (13)	1.0973 (14)	0.3656 (13)	0.034 (5)*	
H12B	0.8548 (13)	1.0425 (13)	0.4541 (13)	0.032 (5)*	
C13	0.74617 (9)	0.69873 (10)	0.33799 (9)	0.0188 (2)	
C14	0.66946 (10)	0.64706 (11)	0.33906 (10)	0.0221 (3)	
H14	0.6147 (14)	0.6514 (14)	0.2963 (13)	0.035 (5)*	
C15	0.68669 (11)	0.58031 (12)	0.41642 (11)	0.0267 (3)	
H15A	0.6842 (14)	0.5155 (14)	0.3967 (13)	0.033 (5)*	
H15B	0.6374 (14)	0.5901 (14)	0.4575 (13)	0.036 (5)*	
C16	0.78647 (11)	0.60903 (12)	0.46348 (10)	0.0259 (3)	
H16A	0.8283 (14)	0.5559 (15)	0.4694 (13)	0.039 (6)*	
H16B	0.7876 (12)	0.6372 (13)	0.5198 (12)	0.024 (5)*	
Ge2	0.72156 (2)	0.67613 (2)	0.73024 (2)	0.01539 (3)	
O5	0.66868 (7)	0.79050 (9)	0.57513 (7)	0.0292 (2)	
O6	0.65130 (9)	0.67107 (9)	0.89028 (8)	0.0319 (3)	
O7	0.90223 (7)	0.71570 (7)	0.83475 (7)	0.02075 (19)	
O8	0.73350 (7)	0.50898 (8)	0.63530 (7)	0.0237 (2)	
C17	0.73712 (9)	0.77827 (10)	0.65076 (9)	0.0172 (2)	
C18	0.80834 (11)	0.83944 (11)	0.65802 (10)	0.0241 (3)	
H18	0.8624 (14)	0.8432 (14)	0.7071 (13)	0.032 (5)*	
C19	0.79407 (11)	0.90342 (12)	0.57842 (11)	0.0266 (3)	
H19A	0.7938 (14)	0.9697 (14)	0.5976 (13)	0.035 (5)*	
H19B	0.8440 (14)	0.8900 (14)	0.5411 (13)	0.037 (6)*	
C20	0.69603 (11)	0.87337 (12)	0.52929 (11)	0.0265 (3)	
H20A	0.6984 (13)	0.8555 (13)	0.4700 (12)	0.027 (5)*	
H20B	0.6490 (14)	0.9218 (15)	0.5329 (13)	0.037 (5)*	
C21	0.64670 (9)	0.72187 (10)	0.81286 (9)	0.0182 (2)	
C22	0.59783 (10)	0.80127 (11)	0.81080 (10)	0.0235 (3)	
H22	0.5888 (14)	0.8425 (14)	0.7631 (13)	0.038 (6)*	
C23	0.56360 (13)	0.81459 (12)	0.89642 (12)	0.0294 (3)	
C24	0.60078 (15)	0.72545 (13)	0.94731 (11)	0.0324 (4)	
H24A	0.5493 (16)	0.6861 (15)	0.9582 (14)	0.045 (6)*	
H24B	0.6453 (16)	0.7397 (16)	1.0018 (15)	0.046 (6)*	
C25	0.84566 (9)	0.64211 (9)	0.79573 (8)	0.0166 (2)	
C26	0.88659 (10)	0.55803 (11)	0.81029 (10)	0.0215 (3)	
H26	0.8613 (14)	0.4975 (15)	0.7893 (13)	0.040 (6)*	
C27	0.98385 (10)	0.56899 (11)	0.86794 (10)	0.0216 (3)	
H27A	1.0330 (14)	0.5386 (14)	0.8448 (13)	0.034 (5)*	
H27B	0.9826 (14)	0.5428 (14)	0.9284 (13)	0.033 (5)*	
C28	0.99617 (10)	0.67601 (11)	0.86798 (9)	0.0203 (2)	
H28A	1.0206 (14)	0.7041 (13)	0.9236 (13)	0.027 (5)*	
H28B	1.0331 (13)	0.6967 (12)	0.8283 (12)	0.022 (5)*	
C29	0.66849 (9)	0.56460 (10)	0.66718 (9)	0.0185 (2)	
C30	0.58084 (10)	0.53090 (11)	0.64952 (10)	0.0236 (3)	
H30	0.5260 (13)	0.5604 (14)	0.6667 (12)	0.032 (5)*	
C31	0.57860 (10)	0.43934 (11)	0.59975 (11)	0.0250 (3)	
H31A	0.5534 (13)	0.3918 (14)	0.6335 (12)	0.033 (5)*	
H31B	0.5415 (15)	0.4461 (15)	0.5397 (14)	0.047 (6)*	
C32	0.68365 (11)	0.42402 (10)	0.59885 (10)	0.0227 (3)	
H23A	0.4953 (16)	0.8203 (15)	0.8901 (14)	0.043 (6)*	
H23B	0.5908 (16)	0.8691 (16)	0.9306 (15)	0.050 (7)*	
H4A	0.8817 (15)	0.7617 (15)	−0.0042 (14)	0.040 (6)*	
H4B	0.9781 (17)	0.7862 (16)	0.0636 (15)	0.052 (7)*	
H32A	0.6975 (14)	0.4156 (15)	0.5409 (14)	0.041 (6)*	
H32B	0.7082 (13)	0.3748 (13)	0.6351 (12)	0.027 (5)*	
H7A	0.4702 (16)	0.9630 (15)	0.1448 (14)	0.048 (6)*	
H7B	0.5180 (15)	0.9528 (15)	0.0576 (14)	0.045 (6)*	

### Tetrakis(4,5-dihydrofuran-2-yl)germane (2) Atomic displacement parameters (Å^2^)

**Table:**

	*U*^11^	*U*^22^	*U*^33^	*U*^12^	*U*^13^	*U*^23^
Ge1	0.01688 (6)	0.01865 (7)	0.01668 (6)	0.00177 (5)	0.00433 (5)	0.00107 (5)
O1	0.0733 (10)	0.0227 (6)	0.0364 (6)	0.0195 (6)	0.0374 (7)	0.0121 (5)
O2	0.0211 (4)	0.0209 (5)	0.0254 (5)	0.0006 (4)	−0.0023 (4)	0.0009 (4)
O3	0.0227 (5)	0.0265 (6)	0.0361 (6)	−0.0032 (4)	0.0055 (4)	−0.0116 (5)
O4	0.0179 (4)	0.0551 (8)	0.0320 (6)	−0.0060 (5)	−0.0026 (4)	0.0239 (6)
C1	0.0215 (6)	0.0198 (6)	0.0192 (6)	0.0030 (5)	0.0070 (5)	0.0028 (5)
C2	0.0319 (7)	0.0198 (7)	0.0325 (8)	0.0051 (6)	0.0171 (6)	0.0063 (6)
C3	0.0369 (8)	0.0197 (7)	0.0323 (8)	0.0067 (6)	0.0161 (7)	0.0023 (6)
C4	0.0546 (11)	0.0231 (8)	0.0308 (8)	0.0133 (8)	0.0265 (8)	0.0067 (6)
C5	0.0195 (5)	0.0217 (7)	0.0169 (5)	0.0028 (5)	0.0047 (4)	0.0005 (5)
C6	0.0259 (6)	0.0211 (7)	0.0245 (7)	0.0041 (6)	0.0039 (5)	0.0028 (5)
C7	0.0270 (7)	0.0315 (9)	0.0276 (7)	0.0090 (7)	0.0034 (6)	0.0087 (6)
C8	0.0192 (6)	0.0334 (8)	0.0217 (6)	0.0038 (6)	0.0021 (5)	0.0010 (6)
C9	0.0202 (6)	0.0205 (6)	0.0190 (6)	−0.0008 (5)	0.0029 (5)	0.0006 (5)
C10	0.0201 (6)	0.0361 (9)	0.0300 (7)	−0.0024 (6)	0.0041 (5)	0.0017 (7)
C11	0.0253 (7)	0.0341 (9)	0.0322 (8)	−0.0095 (7)	−0.0045 (6)	0.0069 (7)
C12	0.0331 (8)	0.0209 (7)	0.0273 (7)	−0.0070 (6)	−0.0007 (6)	−0.0003 (6)
C13	0.0177 (5)	0.0215 (6)	0.0172 (5)	0.0033 (5)	0.0035 (4)	0.0016 (5)
C14	0.0223 (6)	0.0218 (7)	0.0208 (6)	−0.0018 (5)	0.0006 (5)	0.0023 (5)
C15	0.0231 (6)	0.0291 (8)	0.0271 (7)	−0.0021 (6)	0.0028 (5)	0.0074 (6)
C16	0.0218 (6)	0.0314 (8)	0.0240 (7)	0.0004 (6)	0.0030 (5)	0.0088 (6)
Ge2	0.01513 (6)	0.01417 (6)	0.01657 (6)	−0.00089 (5)	0.00231 (4)	−0.00060 (5)
O5	0.0213 (5)	0.0340 (6)	0.0278 (5)	−0.0084 (5)	−0.0062 (4)	0.0122 (5)
O6	0.0454 (7)	0.0298 (6)	0.0246 (5)	0.0172 (5)	0.0168 (5)	0.0083 (5)
O7	0.0192 (4)	0.0148 (5)	0.0254 (5)	0.0014 (4)	−0.0029 (4)	−0.0022 (4)
O8	0.0208 (4)	0.0193 (5)	0.0304 (5)	−0.0021 (4)	0.0036 (4)	−0.0091 (4)
C17	0.0168 (5)	0.0165 (6)	0.0174 (5)	0.0014 (5)	0.0014 (4)	0.0010 (4)
C18	0.0243 (6)	0.0230 (7)	0.0226 (6)	−0.0061 (5)	−0.0016 (5)	0.0029 (5)
C19	0.0234 (6)	0.0243 (7)	0.0299 (7)	−0.0048 (6)	−0.0001 (6)	0.0077 (6)
C20	0.0231 (6)	0.0293 (8)	0.0254 (7)	−0.0026 (6)	0.0005 (5)	0.0105 (6)
C21	0.0173 (5)	0.0186 (6)	0.0189 (6)	−0.0007 (5)	0.0041 (4)	0.0012 (5)
C22	0.0229 (6)	0.0215 (7)	0.0275 (7)	0.0037 (5)	0.0088 (5)	0.0046 (6)
C23	0.0310 (7)	0.0268 (8)	0.0338 (8)	0.0086 (7)	0.0149 (6)	0.0009 (6)
C24	0.0447 (10)	0.0310 (9)	0.0261 (7)	0.0095 (8)	0.0180 (7)	0.0020 (6)
C25	0.0168 (5)	0.0156 (6)	0.0171 (5)	−0.0003 (5)	0.0025 (4)	−0.0008 (4)
C26	0.0219 (6)	0.0165 (6)	0.0242 (6)	0.0014 (5)	−0.0001 (5)	0.0000 (5)
C27	0.0210 (6)	0.0203 (7)	0.0225 (6)	0.0054 (5)	0.0018 (5)	0.0011 (5)
C28	0.0172 (5)	0.0227 (7)	0.0200 (6)	0.0023 (5)	0.0015 (4)	0.0000 (5)
C29	0.0210 (5)	0.0158 (6)	0.0180 (5)	−0.0014 (5)	0.0023 (4)	−0.0008 (5)
C30	0.0205 (6)	0.0193 (7)	0.0293 (7)	−0.0024 (5)	0.0007 (5)	−0.0024 (5)
C31	0.0222 (6)	0.0163 (6)	0.0326 (8)	−0.0011 (5)	−0.0047 (6)	0.0009 (6)
C32	0.0264 (6)	0.0162 (6)	0.0238 (6)	−0.0027 (5)	0.0008 (5)	−0.0022 (5)

### Tetrakis(4,5-dihydrofuran-2-yl)germane (2) Geometric parameters (Å, º)

**Table:**

Ge1—C1	1.9331 (13)	Ge2—C17	1.9370 (13)
Ge1—C5	1.9326 (14)	Ge2—C21	1.9290 (13)
Ge1—C9	1.9299 (14)	Ge2—C25	1.9329 (13)
Ge1—C13	1.9351 (14)	Ge2—C29	1.9353 (14)
O1—C1	1.3776 (17)	O5—C17	1.3806 (16)
O1—C4	1.4592 (19)	O5—C20	1.4641 (19)
O2—C5	1.3852 (17)	O6—C21	1.3816 (17)
O2—C8	1.4649 (17)	O6—C24	1.4624 (19)
O3—C9	1.3797 (17)	O7—C25	1.3877 (16)
O3—C12	1.4595 (18)	O7—C28	1.4619 (16)
O4—C13	1.3822 (16)	O8—C29	1.3840 (17)
O4—C16	1.4603 (19)	O8—C32	1.4592 (17)
C1—C2	1.323 (2)	C17—C18	1.3314 (19)
C2—H2	0.95 (2)	C18—H18	0.97 (2)
C2—C3	1.501 (2)	C18—C19	1.505 (2)
C3—H3A	0.93 (2)	C19—H19A	0.99 (2)
C3—H3B	0.97 (2)	C19—H19B	1.02 (2)
C3—C4	1.518 (2)	C19—C20	1.526 (2)
C4—H4A	0.93 (2)	C20—H20A	0.952 (18)
C4—H4B	1.02 (2)	C20—H20B	0.97 (2)
C5—C6	1.331 (2)	C21—C22	1.326 (2)
C6—H6	0.97 (2)	C22—H22	0.93 (2)
C6—C7	1.511 (2)	C22—C23	1.503 (2)
C7—C8	1.528 (2)	C23—C24	1.529 (2)
C7—H7A	0.96 (2)	C23—H23A	0.97 (2)
C7—H7B	1.05 (2)	C23—H23B	0.97 (2)
C8—H8A	1.00 (2)	C24—H24A	0.97 (2)
C8—H8B	0.899 (18)	C24—H24B	0.97 (2)
C9—C10	1.325 (2)	C25—C26	1.3313 (19)
C10—H10	0.98 (2)	C26—H26	0.96 (2)
C10—C11	1.509 (3)	C26—C27	1.5119 (19)
C11—H11A	1.04 (2)	C27—H27A	0.95 (2)
C11—H11B	0.960 (19)	C27—H27B	1.00 (2)
C11—C12	1.530 (3)	C27—C28	1.531 (2)
C12—H12A	0.94 (2)	C28—H28A	0.946 (19)
C12—H12B	0.951 (19)	C28—H28B	0.930 (18)
C13—C14	1.328 (2)	C29—C30	1.3266 (19)
C14—H14	0.92 (2)	C30—H30	0.974 (19)
C14—C15	1.503 (2)	C30—C31	1.506 (2)
C15—H15A	0.97 (2)	C31—H31A	0.964 (19)
C15—H15B	1.046 (19)	C31—H31B	0.98 (2)
C15—C16	1.532 (2)	C31—C32	1.529 (2)
C16—H16A	0.96 (2)	C32—H32A	0.96 (2)
C16—H16B	0.950 (18)	C32—H32B	0.920 (19)
			
C1—Ge1—C13	109.37 (6)	C21—Ge2—C17	108.03 (6)
C5—Ge1—C1	109.74 (6)	C21—Ge2—C25	108.58 (5)
C5—Ge1—C13	108.78 (6)	C21—Ge2—C29	112.88 (6)
C9—Ge1—C1	107.67 (6)	C25—Ge2—C17	107.92 (5)
C9—Ge1—C5	108.46 (6)	C25—Ge2—C29	106.91 (6)
C9—Ge1—C13	112.79 (6)	C29—Ge2—C17	112.36 (6)
C1—O1—C4	107.12 (11)	C17—O5—C20	106.95 (11)
C5—O2—C8	106.84 (11)	C21—O6—C24	107.04 (12)
C9—O3—C12	106.83 (12)	C25—O7—C28	106.57 (10)
C13—O4—C16	107.29 (11)	C29—O8—C32	107.01 (11)
O1—C1—Ge1	115.90 (10)	O5—C17—Ge2	118.30 (9)
C2—C1—Ge1	130.51 (11)	C18—C17—Ge2	128.10 (11)
C2—C1—O1	113.56 (13)	C18—C17—O5	113.59 (12)
C1—C2—H2	124.1 (12)	C17—C18—H18	125.9 (12)
C1—C2—C3	110.22 (13)	C17—C18—C19	110.14 (13)
C3—C2—H2	125.5 (12)	C19—C18—H18	123.9 (12)
C2—C3—H3A	112.3 (13)	C18—C19—H19A	110.0 (11)
C2—C3—H3B	113.2 (12)	C18—C19—H19B	110.1 (11)
C2—C3—C4	101.79 (13)	C18—C19—C20	101.56 (12)
H3A—C3—H3B	106.4 (17)	H19A—C19—H19B	113.0 (16)
C4—C3—H3A	111.9 (14)	C20—C19—H19A	111.0 (12)
C4—C3—H3B	111.4 (13)	C20—C19—H19B	110.7 (11)
O1—C4—C3	107.18 (12)	O5—C20—C19	107.20 (12)
O1—C4—H4A	109.2 (13)	O5—C20—H20A	107.9 (11)
O1—C4—H4B	106.5 (13)	O5—C20—H20B	106.7 (12)
C3—C4—H4A	111.6 (13)	C19—C20—H20A	110.3 (11)
C3—C4—H4B	111.0 (13)	C19—C20—H20B	111.1 (12)
H4A—C4—H4B	111.1 (18)	H20A—C20—H20B	113.3 (16)
O2—C5—Ge1	117.07 (10)	O6—C21—Ge2	116.77 (10)
C6—C5—Ge1	129.06 (12)	C22—C21—Ge2	129.17 (11)
C6—C5—O2	113.85 (13)	C22—C21—O6	113.68 (13)
C5—C6—H6	127.5 (12)	C21—C22—H22	123.3 (13)
C5—C6—C7	109.74 (14)	C21—C22—C23	110.38 (13)
C7—C6—H6	122.8 (12)	C23—C22—H22	126.3 (12)
C6—C7—C8	101.79 (12)	C22—C23—C24	101.63 (12)
C6—C7—H7A	114.6 (13)	C22—C23—H23A	114.8 (13)
C6—C7—H7B	107.8 (12)	C22—C23—H23B	114.0 (13)
C8—C7—H7A	112.0 (13)	C24—C23—H23A	111.5 (13)
C8—C7—H7B	112.1 (12)	C24—C23—H23B	108.5 (14)
H7A—C7—H7B	108.5 (17)	H23A—C23—H23B	106.3 (17)
O2—C8—C7	107.01 (12)	O6—C24—C23	107.24 (13)
O2—C8—H8A	107.6 (12)	O6—C24—H24A	106.5 (13)
O2—C8—H8B	103.9 (11)	O6—C24—H24B	107.8 (13)
C7—C8—H8A	115.4 (12)	C23—C24—H24A	111.3 (13)
C7—C8—H8B	114.0 (11)	C23—C24—H24B	112.0 (14)
H8A—C8—H8B	108.1 (16)	H24A—C24—H24B	111.7 (18)
O3—C9—Ge1	118.25 (9)	O7—C25—Ge2	116.20 (9)
C10—C9—Ge1	127.58 (12)	C26—C25—Ge2	130.20 (11)
C10—C9—O3	114.16 (14)	C26—C25—O7	113.59 (12)
C9—C10—H10	127.0 (13)	C25—C26—H26	128.1 (12)
C9—C10—C11	109.94 (15)	C25—C26—C27	109.57 (13)
C11—C10—H10	123.1 (13)	C27—C26—H26	122.3 (12)
C10—C11—H11A	111.0 (12)	C26—C27—H27A	113.8 (12)
C10—C11—H11B	109.9 (12)	C26—C27—H27B	108.9 (11)
C10—C11—C12	101.51 (13)	C26—C27—C28	101.32 (11)
H11A—C11—H11B	109.4 (16)	H27A—C27—H27B	108.9 (16)
C12—C11—H11A	111.8 (11)	C28—C27—H27A	110.9 (12)
C12—C11—H11B	113.0 (12)	C28—C27—H27B	113.0 (11)
O3—C12—C11	107.40 (13)	O7—C28—C27	106.50 (11)
O3—C12—H12A	107.2 (12)	O7—C28—H28A	108.0 (12)
O3—C12—H12B	105.2 (12)	O7—C28—H28B	104.8 (11)
C11—C12—H12A	114.5 (12)	C27—C28—H28A	116.2 (12)
C11—C12—H12B	112.1 (12)	C27—C28—H28B	113.2 (11)
H12A—C12—H12B	110.0 (16)	H28A—C28—H28B	107.4 (16)
O4—C13—Ge1	117.16 (10)	O8—C29—Ge2	114.61 (9)
C14—C13—Ge1	129.45 (11)	C30—C29—Ge2	131.68 (11)
C14—C13—O4	113.39 (13)	C30—C29—O8	113.71 (12)
C13—C14—H14	123.4 (13)	C29—C30—H30	125.3 (12)
C13—C14—C15	110.38 (13)	C29—C30—C31	109.97 (13)
C15—C14—H14	126.2 (13)	C31—C30—H30	124.8 (12)
C14—C15—H15A	111.2 (11)	C30—C31—H31A	107.8 (12)
C14—C15—H15B	111.5 (11)	C30—C31—H31B	110.4 (13)
C14—C15—C16	101.60 (12)	C30—C31—C32	101.74 (12)
H15A—C15—H15B	109.1 (15)	H31A—C31—H31B	112.5 (17)
C16—C15—H15A	112.0 (12)	C32—C31—H31A	112.3 (11)
C16—C15—H15B	111.2 (11)	C32—C31—H31B	111.5 (13)
O4—C16—C15	106.89 (12)	O8—C32—C31	106.96 (12)
O4—C16—H16A	108.0 (12)	O8—C32—H32A	106.6 (13)
O4—C16—H16B	108.1 (11)	O8—C32—H32B	107.1 (12)
C15—C16—H16A	110.4 (12)	C31—C32—H32A	114.2 (12)
C15—C16—H16B	113.0 (11)	C31—C32—H32B	111.4 (11)
H16A—C16—H16B	110.2 (16)	H32A—C32—H32B	110.2 (16)
			
Ge1—C1—C2—C3	176.36 (12)	Ge2—C17—C18—C19	176.68 (11)
Ge1—C5—C6—C7	178.01 (11)	Ge2—C21—C22—C23	−170.77 (12)
Ge1—C9—C10—C11	179.83 (11)	Ge2—C25—C26—C27	−178.47 (10)
Ge1—C13—C14—C15	−179.37 (11)	Ge2—C29—C30—C31	−178.54 (11)
O1—C1—C2—C3	−1.5 (2)	O5—C17—C18—C19	−1.66 (19)
O2—C5—C6—C7	−0.57 (18)	O6—C21—C22—C23	1.84 (19)
O3—C9—C10—C11	−0.27 (19)	O7—C25—C26—C27	1.18 (17)
O4—C13—C14—C15	0.09 (19)	O8—C29—C30—C31	0.67 (18)
C1—O1—C4—C3	2.7 (2)	C17—O5—C20—C19	6.77 (18)
C1—C2—C3—C4	3.0 (2)	C17—C18—C19—C20	5.58 (19)
C2—C3—C4—O1	−3.4 (2)	C18—C19—C20—O5	−7.30 (18)
C4—O1—C1—Ge1	−179.00 (13)	C20—O5—C17—Ge2	178.16 (10)
C4—O1—C1—C2	−0.8 (2)	C20—O5—C17—C18	−3.32 (18)
C5—O2—C8—C7	8.45 (15)	C21—O6—C24—C23	0.6 (2)
C5—C6—C7—C8	5.62 (17)	C21—C22—C23—C24	−1.27 (19)
C6—C7—C8—O2	−8.37 (16)	C22—C23—C24—O6	0.35 (19)
C8—O2—C5—Ge1	176.14 (9)	C24—O6—C21—Ge2	172.03 (11)
C8—O2—C5—C6	−5.10 (16)	C24—O6—C21—C22	−1.55 (19)
C9—O3—C12—C11	−4.04 (16)	C25—O7—C28—C27	−15.01 (14)
C9—C10—C11—C12	−2.19 (18)	C25—C26—C27—C28	−10.12 (16)
C10—C11—C12—O3	3.72 (17)	C26—C27—C28—O7	14.94 (14)
C12—O3—C9—Ge1	−177.31 (10)	C28—O7—C25—Ge2	−171.34 (9)
C12—O3—C9—C10	2.78 (18)	C28—O7—C25—C26	8.96 (16)
C13—O4—C16—C15	6.62 (18)	C29—O8—C32—C31	7.81 (15)
C13—C14—C15—C16	3.90 (18)	C29—C30—C31—C32	4.11 (17)
C14—C15—C16—O4	−6.24 (17)	C30—C31—C32—O8	−7.10 (16)
C16—O4—C13—Ge1	175.18 (11)	C32—O8—C29—Ge2	173.88 (9)
C16—O4—C13—C14	−4.35 (19)	C32—O8—C29—C30	−5.48 (17)

### Tetrakis(4,5-dihydrofuran-2-yl)germane (2) Hydrogen-bond geometry (Å, º)

**Table:**

*D*—H···*A*	*D*—H	H···*A*	*D*···*A*	*D*—H···*A*
C31—H31*A*···O7^i^	0.964 (19)	2.60 (2)	3.3279 (18)	132.1 (14)
C23—H23*A*···O4^ii^	0.97 (2)	2.57 (2)	3.530 (2)	168.0 (17)
C4—H4*A*···O7^iii^	0.93 (2)	2.63 (2)	3.398 (2)	140.8 (17)

Symmetry codes: (i) −*x*+3/2, *y*−1/2, −*z*+3/2; (ii) *x*−1/2, −*y*+3/2, *z*+1/2; (iii) *x*, *y*, *z*−1.

## References

[bb1] Allen, F. H., Kennard, O., Watson, D. G., Brammer, L., Orpen, A. G. & Taylor, R. (1987). *J. Chem. Soc. Perkin Trans. 2*, pp. S1–S19.

[bb2] Bauer, J. O. & Strohmann, C. (2014). *Angew. Chem. Int. Ed.* **53**, 720–724. 10.1002/anie.201307826 24402799

[bb4] Bruker (2018). *APEX2* and *SAINT.* Bruker AXS Inc., Madison, Wisconsin, USA.

[bb5] Dolomanov, O. V., Bourhis, L. J., Gildea, R. J., Howard, J. A. K. & Puschmann, H. (2009). *J. Appl. Cryst.* **42**, 339–341.

[bb6] Ertschak, N., Popelis, Û., Nichler, I. & Lukevics, E. (1982). *Zh. Obshch. Khim.* **5**, 1181–1187.

[bb7] Etter, M. C., MacDonald, J. C. & Bernstein, J. (1990). *Acta Cryst.* B**46**, 256–262. 10.1107/s0108768189012929 2344397

[bb8] Evans, D. A., Sweeney, Z. K., Rovis, T. & Tedrow, J. S. (2001). *J. Am. Chem. Soc.* **123**, 12095–12096. 10.1021/ja011983i 11724622

[bb9] Gevorgyan, V., Borisova, L. & Lukevics, E. (1989). *J. Organomet. Chem.* **368**, 19–21.

[bb10] Gevorgyan, V., Borisova, L. & Lukevics, E. (1990). *J. Organomet. Chem.* **393**, 57–67.

[bb11] Gevorgyan, V., Borisova, L. & Lukevics, E. (1992). *J. Organomet. Chem.* **441**, 381–387.

[bb12] Groom, C. R., Bruno, I. J., Lightfoot, M. P. & Ward, S. C. (2016). *Acta Cryst.* B**72**, 171–179. 10.1107/S2052520616003954 PMC4822653 27048719

[bb13] Krause, L., Herbst-Irmer, R., Sheldrick, G. M. & Stalke, D. (2015). *J. Appl. Cryst.* **48**, 3–10. 10.1107/S1600576714022985 PMC4453166 26089746

[bb14] Krupp, A., Barth, E. R., Seymen, R. & Strohmann, C. (2020). *Acta Cryst.* E**76**, 1514–1519. 10.1107/S2056989020011470 PMC7472748 32939310

[bb15] Lazraq, M., Escudié, J., Couret, C., Satgé, J., Dräger, M. & Dammel, R. (1988). *Angew. Chem.* **100**, 885–887.

[bb16] Li, T. & Zhang, L. (2018). *J. Am. Chem. Soc.* **140**, 17439–17443. 10.1021/jacs.8b12478 PMC6535146 30525525

[bb17] Lukevics, E., Gevorgyan, V. & Borisova, L. (1997). *Chem. Heterocycl. Compd.* **33**, 161–163.

[bb18] Lukevics, E., Gevorgyan, V., Rosite, S., Gavaps, M. & Mascheika, I. (1984). *LZA Vēstis*, **1**, 109–111.

[bb19] Lukevics, E., Gevorgyan, V. N., Goldberg, Y. S. & Shymanska, M. V. (1985). *J. Organomet. Chem.* **294**, 163–171.

[bb20] Lukevits, E., Borisova, L. & Gevorgyan, V. (1993). *Chem. Heterocycl. Compd.* **29**, 735–743.

[bb21] Macrae, C. F., Sovago, I., Cottrell, S. J., Galek, P. T. A., McCabe, P., Pidcock, E., Platings, M., Shields, G. P., Stevens, J. S., Towler, M. & Wood, P. A. (2020). *J. Appl. Cryst.* **53**, 226–235. 10.1107/S1600576719014092 PMC6998782 32047413

[bb22] Murakami, M., Hayashi, M. & Ito, Y. (1994). *J. Org. Chem.* **59**, 7910–7914.

[bb23] Neugebauer, P., Klingebiel, U. & Noltemeyer, M. (2000). *Z. Naturforsch. B*, **55**, 913–923.

[bb24] Schmidt, A., Krupp, A., Barth, E. R. & Strohmann, C. (2022). *Acta Cryst.* E**78**, 23–28. 10.1107/S2056989021012548 PMC8739194 35079417

[bb25] Sheldrick, G. M. (2008). *Acta Cryst.* A**64**, 112–122. 10.1107/S0108767307043930 18156677

[bb26] Sheldrick, G. M. (2015*a*). *Acta Cryst.* A**71**, 3–8.

[bb27] Sheldrick, G. M. (2015*b*). *Acta Cryst.* C**71**, 3–8.

[bb28] Spackman, P. R., Turner, M. J., McKinnon, J. J., Wolff, S. K., Grimwood, D. J., Jayatilaka, D. & Spackman, M. A. (2021). *J. Appl. Cryst.* **54**, 1006–1011. 10.1107/S1600576721002910 PMC8202033 34188619

[bb29] Tacke, R., Lopex–Mras, A., Sperlich, J., Strohmann, C., Kuhs, W. F., Mattern, G. & Sebald, A. (1993). *Chem. Ber.* **126**, 851–861.

[bb30] Tacke, R., Sperlich, J., Strohmann, C. & Mattern, G. (1991). *Chem. Ber.* **124**, 1491–1496.

[bb31] Westrip, S. P. (2010). *J. Appl. Cryst.* **43**, 920–925.

